# A multiscale cell‐based model of tumor growth for chemotherapy assessment and tumor‐targeted therapy through a 3D computational approach

**DOI:** 10.1111/cpr.13187

**Published:** 2022-02-07

**Authors:** Sahar Jafari Nivlouei, Madjid Soltani, Ebrahim Shirani, Mohammad Reza Salimpour, Rui Travasso, João Carvalho

**Affiliations:** ^1^ 48456 Department of Mechanical Engineering Isfahan University of Technology Isafahan Iran; ^2^ Department of Physics CFisUC University of Coimbra Coimbra Portugal; ^3^ Department of Mechanical Engineering K. N. Toosi University of Technology Tehran Iran; ^4^ Department of Electrical and Computer Engineering University of Waterloo Waterloo ON Canada; ^5^ Centre for Biotechnology and Bioengineering (CBB) University of Waterloo Waterloo ON Canada; ^6^ Advanced Bioengineering Initiative Center Computational Medicine Center K. N. Toosi University of Technology Tehran Iran; ^7^ Cancer Biology Research Center Cancer Institute of Iran Tehran University of Medical Sciences Tehran Iran; ^8^ Department of Mechanical Engineering Foolad Institute of Technology Fooladshahr Iran

**Keywords:** cellular Potts model, chemotherapy, computational biology, signaling transduction, targeted therapy, tumor‐induced angiogenesis

## Abstract

**Objectives:**

Computational modeling of biological systems is a powerful tool to clarify diverse processes contributing to cancer. The aim is to clarify the complex biochemical and mechanical interactions between cells, the relevance of intracellular signaling pathways in tumor progression and related events to the cancer treatments, which are largely ignored in previous studies.

**Materials and Methods:**

A three‐dimensional multiscale cell‐based model is developed, covering multiple time and spatial scales, including intracellular, cellular, and extracellular processes. The model generates a realistic representation of the processes involved from an implementation of the signaling transduction network.

**Results:**

Considering a benign tumor development, results are in good agreement with the experimental ones, which identify three different phases in tumor growth. Simulating tumor vascular growth, results predict a highly vascularized tumor morphology in a lobulated form, a consequence of cells' motile behavior. A novel systematic study of chemotherapy intervention, in combination with targeted therapy, is presented to address the capability of the model to evaluate typical clinical protocols. The model also performs a dose comparison study in order to optimize treatment efficacy and surveys the effect of chemotherapy initiation delays and different regimens.

**Conclusions:**

Results not only provide detailed insights into tumor progression, but also support suggestions for clinical implementation. This is a major step toward the goal of predicting the effects of not only traditional chemotherapy but also tumor‐targeted therapies.

## Graphical abstract

The model simulates the tumor development processes at intracellular, cellular, and extracellular scales. A diagram of the different events at each scale is shown. The extracellular scale describes tumor growth associated with angiogenesis, by calculating the diffusion of VEGF, nutrients, and the therapeutic drugs. The environmental conditions activate receptors' prompting signaling cascades inside cells, and, consequently, the behavior of each cell is predicted at the intracellular scale. The interaction between cells of different phenotypes is described at the cellular level.

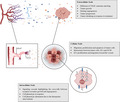

## 1 INTRODUCTION

Cancer is characterized by the aberrant properties of tumor cells, including abnormal fast growth and division as well as the resistance to apoptosis. The initiation and development of cancer often depend on a series of genetic mutations affecting cellular programs. This leads to the effort by the scientific community on identifying the molecular basis of cancer and on the development of mathematical and computational approaches addressing tumor morphological evolution. The main goal of such models is to describe the observed phenomena based on the biological mechanisms that control the system's behavior. Moreover, the correct implementation in these simulations of the main physical mechanisms underlining tumor development leads to a real possibility for evaluating pre‐clinical drug design opportunities and helping the optimization of drug delivery.^1^, ^2^

The development of benign tumors is caused by excessive cell proliferation, which is commonly limited by space, or more usually, by the nutrient availability in the tissue. This early phase of tumor development, labeled avascular growth, has tumor cells in the hypoxic cell state, where they are able to survive with a lower nutrient concentration. Hypoxic cells within a tumor express the hypoxia‐inducible factor‐1 (HIF‐1), upregulating pro‐angiogenic factors and triggering tumor vascularization. A denser vasculature gives access to an additional supply of nutrients, driving the tumor growth to the vascular phase. Tumor vascular growth is a feature of malignancy, enabling tumor cells to invade other tissues by entering the circulation via nearby blood vessels (metastasis).^3^, ^4^

Various mathematical techniques have been used to simulate tumor growth and associated processes, being applied to tumors both in the avascular^5^, ^6^, ^7^ and at the vascular stages.^8^, ^9^, ^10^, ^11^ Indeed, most of the early mathematical models of tumor growth address avascular tumor morphology. Developing mathematical models of tumor‐induced angiogenesis permits a more realistic description of nutrient availability in tumors.^12^, ^13^, ^14^, ^15^ See^16^, ^17^, ^18^, ^19^ for complete reviews of angiogenesis models. Recently, multiscale approaches have been introduced to reproduce the biological and physical mechanisms in tumor growth and angiogenesis. These models consider both subcellular and tissue scales.^19^, ^20^, ^21^, ^22^, ^23^

Alarcón et al.^24^, ^25^, ^26^ introduced a hybrid structured lattice‐based model to simulate vascular tumor growth, which considers blood flow and oxygen transport in a tissue scale and accounts for cellular interactions and progress in cellular and intracellular scales. The model investigates the effects of nutrient spatial heterogeneity on the evolution and invasion of cancerous tissue, and the emergent growth laws. The diffusive transport of oxygen and VEGF within the tissue is described through reaction–diffusion equations. In a subsequent study, the authors considered the diffusion of standard cytotoxic drugs as a treatment and investigated the effects of vessel normalization on chemotherapy.^26^ The authors concluded that vessel normalization improves the efficiency of chemotherapeutic drugs, observing also the decrease in the prevalence of hypoxia. Subsequently, Owen et al.^27^ developed the same model into a more realistic one by simulating blood flow and vascular remodeling during angiogenesis. Their findings show that a tumor may continue to grow near the parent vessel until the formation of new vessels creates bridges between adjacent vessels. Following these studies, a 3D version of the proposed model is described by Perfahl et al.^28^ A more recent study presented by Stepanova et al. introduced a hybrid stochastic 2D multiscale model which accounts for cell rearrangements in the formation of angiogenic networks. The authors stated that their model reproduces properties describing the gene expression patterns of ECs. The results predict that there is an imbalance between effective sprout elongation and branching when cells at the sprouts have difficulty in rearranging their position.^29^ Stéphanou et al.^30^, ^31^ simulated tumor development and angiogenesis with a hybrid model. This uses a lattice‐based cellular automaton (CA) to represent cells and their interaction and a continuous PDE describing the evolution of endothelial cells density. This model explores the alterations of the vessels and their effect on tumor dormancy. The described results show tumor dormancy as a consequence of vascular changes in the larger upstream vessels in the host tissue. Welter et al.^32^ presented a different hybrid lattice‐based model for trans‐vascular oxygen transport in a synthetic tumor and host tissue vasculature using a series of steady‐state diffusion equations. Reduction in the vessels' radii leads to a decrease in blood oxygen saturation in tumors in comparison with normal tissue, which is interpreted as emulating vessels' compression caused by intra‐tumoral stress.

Cell‐based models have a great potential in tracking single‐cell traits and cell behavior rules. The cellular Potts model (CPM) is a widely used cell‐based modeling framework, simulating biophysical and molecular interactions between cells, based on biophysical cellular properties. This capability makes it a popular approach to describe events in cancer development. Shirinifard et al.^33^ used a 3D multiscale cellular Potts model to study tumor growth and angiogenesis. The tumor cell behavior is determined by oxygen concentration in the microenvironment, diffusing from the blood vessels. Szabo et al.^34^ introduced a hybrid model that couples CPM with a continuous tissue scale, to describe the concentration of oxygen, glucose, and lactate. This model successfully predicted the effect of vessel blocking probabilities on the evolution of tumor cells. Accordingly, instabilities in blood supply can lead to a reduction in tumor aggressiveness. Kanigel Winner et al.^35^ used the CPM framework to investigate the administration of anticancer drugs to ovarian cancers. The model provides a comparison between the effects of intravenous injections and intraperitoneal infusions in tumor penetration. The authors reported that intraperitoneal infusion is the preferable route in the initial growth phase, when the tumor is still small and avascular. Jafari Nivlouei and co‐workers^36^ proposed a 2D multiscale agent‐based model, addressing two distinct phases in tumor growth. In each stage, tumor progression is considered with and without normal healthy cells. The authors reported the formation of a dense intra‐tumoral vascular network throughout the entire tumor mass as a sign of a high malignancy grade.

In what concerns cancer therapy, computational studies focusing on tumor response to therapy are fundamental tools that facilitate the understanding of drug's mechanism of action, helping to determine the most effective treatment protocols. Different approaches to chemotherapy modeling have been proposed, including continuous^37^ and hybrid discrete‐continuum models, in which the model describes the effect of interstitial fluid pressure and lymphatic drainage on drug delivery to tumors.^38^, ^39^, ^40^ This class of studies permits the evaluation of the parameters that limit the delivery of nutrients and therapy. More recently, multicellular and multiscale techniques, which incorporate drug therapy at the extracellular level, are becoming increasingly important in tumor treatment simulation. Wang et al.^41^ developed a multiscale agent‐based model to simulate the melanoma tumor vascular growth and to study the response of tumor to combined drugs treatments. The authors reported that the interruption in the communications between melanoma cells and the vasculature might increase the drugs' effectiveness.

Targeted therapy is a novel type of treatment which reduces systemic drug toxicity by inducing modifications in the tumor microenvironment and not in normal cells.^42^, ^43^ Targeted drugs are characterized by the binding of their therapeutic molecules to specifically expressed receptors on the tumor cells' membranes. Kim and co‐workers^44^ modeled targeted therapy by focusing on specific intracellular signaling pathways that prevent cancer cells' abnormal behavior and finally induce cell apoptosis or suppress cell growth. They developed a hybrid model where they targeted the MAPK and PI3K‐AKT signaling pathways, which are activated in lung cancer, and used it to investigate the effects of this pathway inhibition under different microenvironmental conditions. The model uses a CA approach to describe the cellular process and a set of ODEs to address tumor response to the targeted therapy. The authors suggested a new treatment combination strategy based on the predicted cell signaling responses. More recently, a new class of targeted therapies has been developed, targeting cells in the hypoxic regions. In hypoxia‐activated pro‐drugs (HAPs), the cytotoxic agents are released under low oxygen pressure.^45^ Hong et al.^46^ introduced a hybrid model that combines the CA model with continuous transport equations to simulate tumor response to HAP and to explore the bystander effects of the therapy. A similar study was presented by Karolak et al.,^47^ using a model that combines a discrete model with advection–diffusion–reaction equations describing the concentrations of oxygen and drug.

Here, a 3D multiscale model is developed to cover multicellular dynamics of tumor growth and tumor‐induced angiogenesis. This work extends the 2D model proposed by Jafari Nivlouei et al.^36^ that is the first to consider the cellular interactions and cell behavior in tumor progression process as a consequence of the activation of oncogenes and the deactivation of gene signaling pathways. The current model involves different scales, including intracellular, cellular, and extracellular. It describes the mechanical interactions between cells, based on biochemical mechanisms, to generate realistic predictions. At the intracellular scale, the cell phenotype is determined directly from the signaling pathways' gene regulatory network, and alterations in the cells' response to different receiving signals are investigated. At the cellular scale, the model uses the cell‐based cellular Potts model to simulate tumor progression, describing the interactions between different cells' types and with their microenvironment. In this work, the mechanical environment applied to each cell determines its dynamics. To model the formation of new vessels, the local concentration of vascular endothelial growth factor (VEGF) diffused from the tumor, along with vessel‐supplied nutrients, is calculated in the extracellular scale from partial differential equations (PDEs). Similarly, in order to describe the response of tumor cells to chemotherapy, cytotoxic drug pharmacodynamics is modeled through a set of PDEs. This study aims to link models of avascular and vascular tumor growth as a predictive model of carcinogenesis, to mimic experimental assays and test different therapeutic strategies, including chemotherapy and targeted therapy. Despite remarkable progress in the development of models of tumor growth and angiogenesis over the last three decades, previous mathematical investigations have largely ignored the complex biochemical and mechanical interactions between cells in the host microenvironment, and the relevance of intracellular signaling pathways in tumor progression and related events to the cancer treatments. The current model presents an explicit description of the key interactions that mediate morphogenic processes and highlights receptor influence in cell state evolution and extracellular reaction–diffusion dynamics. This provides a significant and novel contribution to the field of simulating tumor growth and different methods of cancer treatment in a simplified way. The model integrates all the information received from each spatial and temporal scale to predict the system response. It enables us to survey cell phenotypic alterations by considering the interaction of signaling molecules and the signaling pathways. This helps to explore the mechanism of anti‐tumor and ECM‐targeted strategies by inhibiting the activity of specific receptors. Results will not only provide detailed insights into tumor progression, but the model is also a step toward clinical implementation. This represents an opportunity to analyze tumor response to both treatment strategies (i.e., chemotherapy and combination therapy) and to evaluate typical clinical protocols.

## 2 MATERIAL AND METHODS

The model simulates the tumor development processes at intracellular, cellular, and extracellular scales. Each scale of the model is presented in the following sections to detail the implemented mechanisms of tumor progression.

### 2.1 Intracellular scale

At the intracellular level of this tumor growth simulation, signal transduction pathways determine the cellular processes occurring during tumor development and angiogenesis. Intracellular signaling leads to genetic activity modulation in the cancer cells and to the production of growth factors, which increase the cell proliferation rate, promote its survival, and facilitate healthy tissue invasion. To investigate the mechanisms by which a cell responds to the environmental signals and, consequently, drives cancer initiation and development, the model focuses on pivotal pathways involved in various types of cancer including: receptor tyrosine kinases (RTKs), integrin, cadherin and Wnt.

Oncogenic mutations not only cause the overexpression of genes, but also can produce mutated proteins. Growth factor receptor tyrosine kinases (RTKs) are often de‐regulated in neoplasms. These are proteins involved in commonly activated survival signaling pathways, whose activity leads to stimulation of serine/threonine kinases (e.g., Raf and Akt) and lipid kinases (e.g., PI3Ks) through the activation of small GTPases (e.g., Ras). Moreover, the activation of Ras can originate from the loss of neurofibromin (NF1) protein, encoded by the NF1 gene. NF1 functions as a tumor suppressor that negatively regulates the activity of Ras.^48^, ^49^, ^50^, ^51^ Loss of tumor suppressors' function results in cancer initiation and progression because of their role in cell division inhibition, induction of apoptosis, and metastasis suppression. While the hyperactivation of Ras‐ERK and PI3K‐Akt signaling pathways can lead to excessive proliferation in tumor cells, mutations can promote the cancer phenotype by disabling cell death signaling.^52^, ^53^ For instance, p53 is known as a tumor suppressor protein whose loss through mutation can contribute to tumor development by the interruption of cell death signaling, as it regulates cell apoptosis by binding directly to Bax, a pro‐apoptotic protein.^54^ As tumor progression transits into the malignant phase, cells are more aggressive and can migrate and invade the surrounding tissue. Cells' migration can be regulated by different stimuli, such as growth factors and adhesion receptors.^55^, ^56^ Importantly, integrin receptors and matrix adhesion proteins (e.g., FAK), accompanied by cadherin cell–cell adhesion complexes, are known as major targets that regulate various cellular functions, including cell survival as well as cell migration through downstream effectors.^57^, ^58^ Integrins are transmembrane receptors that mediate cells' adhesion to the extracellular matrix (ECM). Integrin attachment to the ECM deregulates the activation of the mitogen‐activated protein (MAP) kinase cascade that controls cell cycle progression and drives the actin cytoskeleton, which is fundamental for cell motility.^59^, ^60^, ^61^

Cell–cell adhesion through the E‐cadherin transmembrane receptor keeps the cells together and guarantees the formation of cohesive multicellular structures, promoting cell viability in multicellular organisms.^62^, ^63^, ^64^ E‐cadherin provides a mechanism for cell communication through cell–cell junctions and mediates contact inhibition of cell growth.^65^, ^66^ Furthermore, the endothelial cell (EC)‐specific cadherin transmembrane receptor, VE‐cadherin, whose association with the protein ß‐catenin facilitates its binding to the actin cytoskeleton, is responsible for the tight but dynamic connection between neighboring cells.^67^ This protein plays an important role in providing a cohesive structure for the new blood vessel. Strikingly, the cadherin–catenin adhesion system regulates cell proliferation and migration through downstream signaling effects during cancer development. E‐cadherin loss of expression promotes the release of ß‐catenin into the cytosol, which results in the activation of Wnt signaling. ß‐catenin is the main effector of the Wnt signaling pathway,^68^, ^69^ and E‐cadherin negatively regulates the Wnt/ß‐catenin signaling. Nevertheless, the loss of expression of cadherin by itself is not sufficient for the activation of ß‐catenin signaling.^70^ Recent research reveals that the loss of APC function is associated with increased levels of ß‐catenin.^71^, ^72^ APC is a tumor suppressor localized inside the cells' nucleus, which regulates cell proliferation by inhibiting Wnt/ß‐catenin signaling, and facilitates cell apoptosis to suppress tumor progression and metastatic cell spread.^73^ The reintroduction of APC into mutant cells, in order to restore its function in Wnt/ß‐catenin signaling, has been investigated in several therapeutic treatments.^74^, ^75^, ^76^ Experimental observations demonstrate that the early loss of APC function and the activation of ß‐catenin can be followed by the later loss of E‐cadherin, leading to the cell's invasive behavior.^70^

**FIGURE 1:**
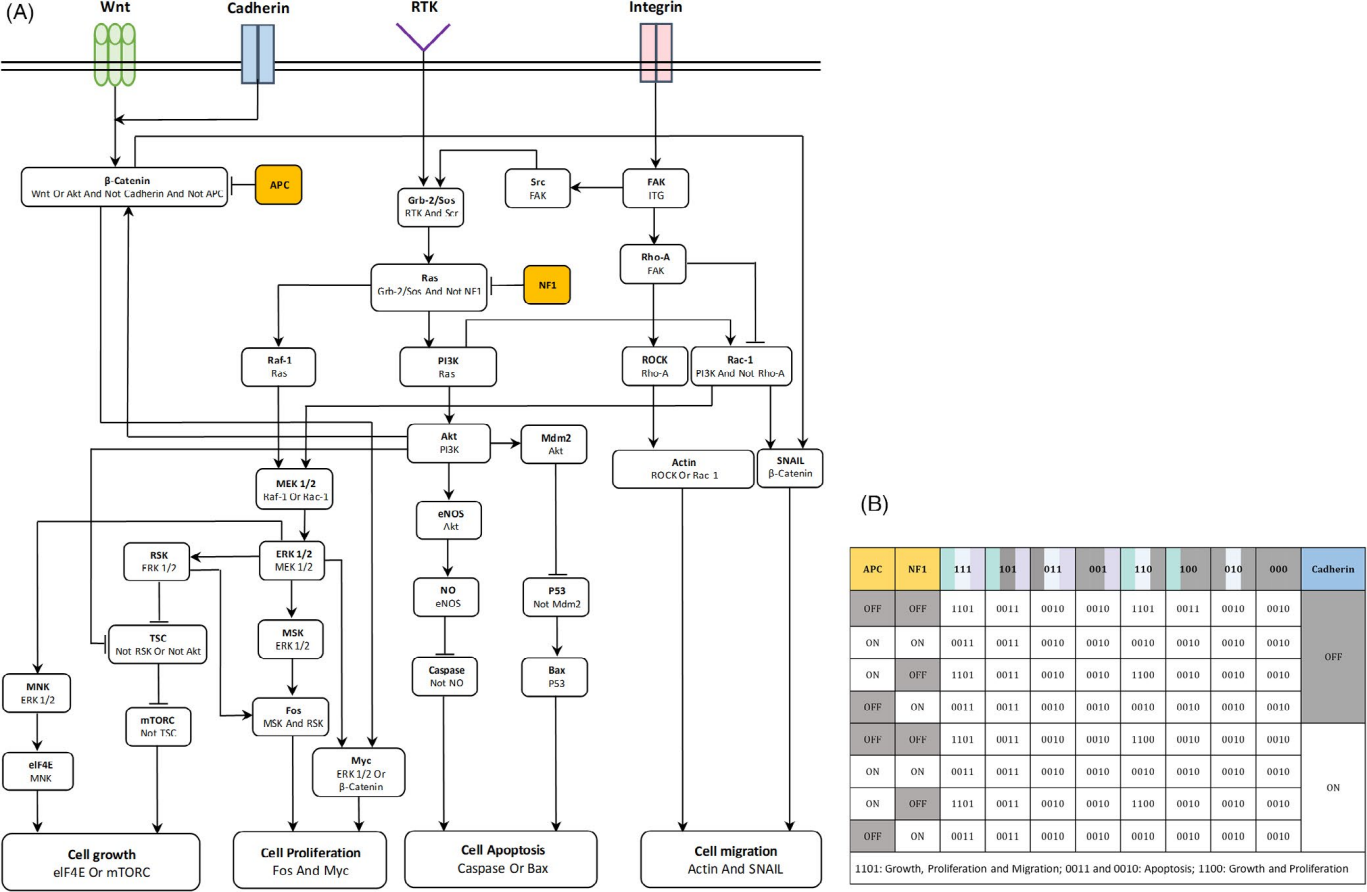
Determination of cell phenotype at the intracellular level. (A) Signal transduction network of the current tumor growth model, focusing on Ras‐PI3K‐Akt and Wnt/ß‐catenin signaling pathways. External stimuli, Wnt, RTK, cadherin, and integrin, are considered to regulate the cell cycle progression and to determine the cell's phenotype. Arrows represent the activation of the protein involved and bar‐headed lines describe inhibition. Different experimental studies are used to integrate the information of the most important effectors that play a key role in cell cycle regulation, as presented in Table 1 (Methods and Materials). (B) Boolean map of cell phenotype for different input configurations. Colors indicate activity of the signal from each correspondent receptor (i.e., integrin, RTK, and Wnt), and receptor inactivation is shown in gray. For instance, considering cadherin activation, case (110) represents receiving signal from integrin and RTK, and no signal from Wnt, and the model predicts cell growth and proliferation (1100). The binary codes on the first row specify the integrin, RTK, and Wnt states, respectively

Considering the described key events in tumor growth and angiogenesis, a signaling cascade is modeled based on the cross talk between the main regulators of growth factors (RTKs), integrin, cadherin, and Wnt (Figure 1A). Moreover, different experimental studies are used to integrate the information of the most important effectors that play a key role in cell cycle regulation, as presented in Table 1. The dependences between the network nodes are specified by the arrows, which indicate the activation of the corresponding effector. On the contrary, an inhibitory effect is pictured as bar‐headed lines. The aim of modeling the intracellular scale is to determine the cell phenotype in response to the active signals.

**TABLE 1:** Boolean dependence relations of the nodes presented in the signaling network of Figure 1A, based on the experimental data of given references. The colors correspond to the color coding of the nodes in Figure 1A

Node	Dependence relation	Reference
Integrin	External signal (Integrin binding)	77
RTK	External signal (VEGF binding)	78
E‐cadherin	External signal (cadherin binding)	79, 80
Wnt	External signal (Frizzled receptor)	81, 82
ß‐Catenin	Wnt Or Akt And Not cadherin AND Not APC	79, 83
APC	External signal	84
Grb‐2/Sos	RTK And Scr	85
Src	FAK	86, 87
FAK	ITG	87
Rho‐A	FAK	87
ROCK	Rho‐A	88, 89
Rac‐1	PI3K And Not Rho‐A	90, 91
Ras	Grb‐2/Sos And Not NF1	85, 92, 93
NF1	External signal	48, 49, 50
Raf‐1	Ras	94, 95, 96
MEK1/2	Raf‐1 Or Rac‐1	94, 97
ERK1/2	MEK1/2	94, 97
RSK	ERK 1/2	98, 99
TSC	Not RSK Or Not Akt	99, 100
mTORC	Not TSC	101
MNK	ERK 1/2	102
eIF4E	MNK	103
MSK	ERK 1/2	104
Fos	MSK And RSK	105, 106
Myc	ERK 1/2 Or ß‐Catenin	107, 108
PI3K	Ras	94
Akt	PI3K	109, 110
eNOS	Akt	111
NO	eNOS	112
Caspase	Not NO	110
Mdm2	Akt	54
p53	Not Mdm2	113
Bax	p53	114
Actin	ROCK Or Rac‐1	91
SNAIL	ß‐Catenin	82
Cell growth	eIF4E Or mTORC	101, 103
Cell proliferation	Fos And Myc	94, 98, 115
Cell apoptosis	Caspase Or p53	54, 74
Cell migration	Actin And SNAIL	82, 91

Our model is implemented as a Boolean network model that integrates the signaling network and, from its output, determines the cell phenotype. Quantitative information for the kinetics of the relevant biochemical reactions is scarce and imprecise, and the initial state of the nodes under different circumstances is almost inaccessible. Stochastic methods are commonly applied to simulate biological systems and handle the lack of data.^116^, ^117^, ^118^, ^119^ Boolean network approaches were introduced by Stuart Kauffman^120^, ^121^ and have become a useful tool to examine the dynamics of gene regulatory networks. They are also used to predict unknown correlations inside the signaling networks, as in recent studies on angiogenesis.^122^, ^123^ Hence, using a MATLAB‐based toolbox, a Boolean network model has been applied to describe the proposed signaling cascade, critical to determining the cellular phenotype during tumor growth. For a more detailed description of Boolean network models, see references.^124^, ^125^, ^126^

### 2.2 Cellular scale

To capture system dynamics at the cellular level, a lattice‐based Monte Carlo model, a cellular Potts model, is extended to describe the interactions between cells and the ECM in three dimensions. The cellular Potts model is a stochastic model developed by Glazier and Graner,^127^ which describes cells' behavior in response to environmental cues based on the effective energy and constraints. This approach enables us to incorporate the intracellular scale, capturing both tumor and vessel cells' interactions as they are growing, proliferating, migrating, or undergoing apoptosis.

The model includes a list of generalized cells (i.e., cancer cells, endothelial cells, and ECM) that are spatially extended through the computational domain and reside on a three‐dimensional cell lattice. Each cell type has a unique cell index, *τ*, which is assigned to every entity occupying a lattice site. One unique cell index tag, for example, *σ* = 1, 2, …, represents each individual cell. The “cell index” 0 is assigned to all lattice sites that are filled by ECM. Lattice site occupation evolves based on a total effective energy minimization algorithm, which means that any configuration evolves toward one that satisfies the energy minimization. The term representing energy is denominated Hamiltonian, H, and the simulations are performed using the Metropolis criteria based on the variation of the Hamiltonian value, ΔH. Accordingly, any change that decreases the total energy is accepted; otherwise, the Boltzmann probability determines the acceptance of the alteration, with the expression: $e^{‐\Delta H/T_{m}}$, where $T_{m}$ is a gauge of system disorder, representing the amplitude level of cell membrane fluctuations, describing the effective cell motility.

In the current study, the Hamiltonian is considered as a sum of four effective energy terms describing cellular adhesion, cell growth, chemotaxis, and guaranteeing cell continuity.

- **The adhesion energy:** describes the interaction between adjacent cells and the cells' contact with the ECM. Depending on the cell type, $J_{\tau ,\tau^{\prime }}$ is the measure of adhesion strength between the entities of types τ and $\tau^{\prime }$ (the larger $J_{\tau ,\tau^{\prime }}$, the weaker the adhesion between types τ and $\tau^{\prime }$)

(1) $$E_{\text{adhesion}}=\sum_{\text{site}}J_{\tau ,\tau^{\prime }}\left(1‐\delta_{\sigma ,\sigma^{\prime }}\right)$$

The sum is run over all the neighboring pixels, σ and $\sigma^{\prime }$ are the cells' ID, and *δ* is the Kronecker symbol.

- **Cell growth:** describes the energy involved in cell growth and proliferation through mitosis. During the cell cycle progression, cells grow until they reach twice their initial volume, and then they divide. After mitosis, the parent cell target volume is restored to its initial volume, and the offspring cell will inherit the type and target volume of the parent cell, being assigned a new unique ID. The energy responsible for setting the target cell size in the Hamiltonian is:

(2) $$E_{\text{growth}}=\sum_{\text{cell}}\gamma_{e}{\left(v_{\sigma }‐V_{\sigma }^{T}\right)}^{2}$$

where $v_{\sigma }$ denotes the cell volume, while $V_{\sigma }^{T}$ is the target volume and $\gamma_{e}$ is the cell elasticity.

- **Chemotaxis:** tumor cells' migration in the microenvironment via chemotaxis is critical in cancer metastasis. Assuming that nutrients and oxygen diffused from vessels work as chemoattractants, tumor cells move toward the vessels and are able to invade distant organs after crossing into the blood stream. The energy involved in the chemotaxis of tumor cells with a migration phenotype is proportional to the concentration gradient of the nutrients (*n*), as:

(3) $$E_{\text{chemotaxis}}=\sum_{\text{cell}}\chi_{\sigma }\Delta n$$

The migration of endothelial cells (ECs) is the most important event that occurs during sprouting angiogenesis. Considering VEGF as a chemoattractant agent that regulates the vascular network development, ECs move toward a higher concentration of VEGF (*V*).

(4) $$\Delta E_{\text{chemotaxis}}=\sum_{\mathrm{cell}}\chi_{\sigma }\Delta V$$

$\chi_{\sigma }$ is a negative parameter that determines the chemotaxis intensity and is denoted as chemotactic potential.

- **Haptotaxis**: describes the movement of cells by adhesion gradients, and in particular cell motion according to the concentration gradient of relevant molecules linked to the ECM.^20^ This mechanism is modeled through the adhesion energy between cells and ECM.
- **Cell continuity:** Cells are a continuous medium. To keep the continuity of lattice sites that are occupied by a single cell, a constraint term is added to the Hamiltonian. This term introduces a severe increase in the system total energy when a cell is about to rupture:

(5) $$E_{\text{continuity}}=\sum_{\text{cell}}\alpha \left(1‐\delta_{v_{\sigma },v_{\sigma }^{\prime }}\right)$$

where α is a large penalty factor that increases the system energy when there is a difference between the current contiguous cell size ($v_{\sigma }$) and the number of lattice sites occupied by the cell with unique identification σ ($v_{\sigma }^{\prime }$).

Therefore, the contribution of the energy terms related to cell adhesion, volume, continuity, and chemotaxis, which is referred collectively as Hamiltonian, will be as follows:

(6) $$H=E_{\text{adhesion}}+E_{\text{growth}}+E_{\text{continuity}}+E_{\text{chemotaxis}}=\sum_{\text{site}}J_{\tau ,\tau^{\prime }}\left(1‐\delta_{\sigma ,\sigma^{\prime }}\right)+\sum_{\text{cell}}\gamma_{e}{\left(v_{\sigma }‐V_{\sigma }^{T}\right)}^{2}+\sum_{\text{cell}}\alpha \left(1‐\delta_{a_{\sigma },a_{\sigma }^{\prime }}\right)+\sum_{\text{cell}}\chi_{\sigma }C$$

where *C* is the chemoattractant agent, replaced by the nutrient concentration *n* in what concerns tumor cells, or the VEGF concentration (*V*) for the activated endothelial cells.

### 2.3 Extracellular scale

At this level, concentrations of nutrients and growth factor, and the drug distribution are described by a set of reaction–diffusion PDEs.

- **Diffusion of nutrients in the ECM**: nutrient and oxygen deprivation (hypoxia) in tumor cells leads to the secretion of angiogenic factors, such as VEGF. This factor activates ECs and stimulates the growth of new capillaries to irrigate the tumor lesion and increase the oxygen supply.^128^ To model this process, a reaction–diffusion equation is used to describe the diffusion, production, and consumption of nutrients from vessels into the microenvironment (Equation 7).

(7) $$\frac{\partial n}{\partial t}=D_{n}\nabla^{2}n‐B\left(x,y,z,n\right)+S_{n}$$

where *n* denotes nutrient concentration, $D_{n}$ is its diffusion coefficient, $S_{n}$ refers to the process of nutrients release from vessels, and *B* is a function expressing the uptake rate of nutrients by tumor cells, as described below:

$$B\left(x,y,z,n\right)=\left\{\begin{array}{ccccc} n & \text{if} & 0\leq n\leq \beta & \text{and} & \left\{\left(x,y,z\right)\subset \text{Cancer cell}\right\}\\ \beta & \text{if} & n>\beta & \text{and} & \left\{\left(x,y,z\right)\subset \text{Cancer cell}\right\}\\ 0 & \text{if} & \; & \; & \left\{\left(x,y,z\right)⊄\text{Cancer cell}\right\} \end{array}\right.$$

where *β* is the maximum consumption rate of nutrients per cell voxel for a tumor cell. The release rate of nutrients from the endothelial cells is given by:

$$S_{n}\left(x,y,z\right)=\left\{\begin{array}{ccc} s_{n} & \text{if} & \left\{\left(x,y,z\right)\subset \text{Endothelial cell}\right\}\\ 0 & \text{if} & \left\{\left(x,y,z\right)⊄\text{Endothelial cell}\right\} \end{array}\right.$$

- **Diffusion of VEGF in the ECM:** The tumor starts to secret VEGF to extend new capillaries by activating the ECs. Hence, a concentration gradient between the tumor and the nearby vascular network is generated, which drives activated ECs to migrate toward the tumor. To simulate sprout migration, VEGF distribution in the domain is governed by a PDE similar to nutrients diffusion. Hence, considering diffusion, uptake, and decay of the VEGF, the equation for VEGF concentration is given by:

(8) $$\begin{array}{cc} \frac{\partial V}{\partial t} & =D_{V}\nabla^{2}V‐k_{V}V‐E\left(x,y,z,V\right)+S_{V}\\ E\left(x,y,z,V\right) & =\left\{\begin{array}{ccccc} V & \text{if} & 0\leq V\leq e & \text{and} & \left\{\left(x,y,z\right)\subset \text{Endothelial cell}\right\}\\ e & \text{if} & V>e & \text{and} & \left\{\left(x,y,z\right)\subset \text{Endothelial cell}\right\}\\ 0 & \text{if} & \; & \; & \left\{\left(x,y,z\right)⊄\text{Endothelial cell}\right\} \end{array}\right.\\ S_{V}\left(x,y,z\right) & =\left\{\begin{array}{ccc} s_{V} & \text{if} & \left\{\left(x,y,z\right)\subset \text{Hypoxic cancer cell}\right\}\\ 0 & \text{if} & \left\{\left(x,y,z\right)⊄\text{Hypoxic cancer cell}\right\} \end{array}\right. \end{array}$$

where $D_{V}$ is the diffusion constant of VEGF (*V*), *k_V_* refers to its decay rate, $S_{V}$ is the function of secretion rate of VEGF, and *E* denotes the uptake function of VEGF by ECs, with a maximum rate of *e*.

- **Diffusion of the chemotherapeutic drug:** The model includes chemotherapy with a cytotoxic drug. The diffusion of drugs from the vessels and the new tumor‐induced capillaries (neovasculature) is described by a reaction–diffusion equation:

(9) $$\frac{\partial c}{\partial t}=D_{c}\nabla^{2}c‐k_{c}c‐R\left(x,y,z,c\right)+S_{c}$$

where *c* denotes the chemotherapy drug concentration, $D_{c}$ its diffusion coefficient, and *k_c_* represents the drug decay rate. *R* is the function of drugs' uptake rate by tumor cells, as described below:

$$R\left(x,y,z,c\right)=\left\{\begin{array}{ccccc} c & \text{if} & 0\leq c\leq \rho & \text{and} & \left\{(x,y,z)\subset \text{Proliferative}\;\text{Cancer cells}\right\}\\ \rho & \text{if} & c>\rho & \text{and} & \left\{(x,y,z)\subset \text{Proliferative}\;\text{Cancer cells}\right\}\\ 0 & \text{if} & \; & \; & \left\{(x,y,z)⊄\text{Proliferative}\;\text{Cancer cells}\right\} \end{array}\right.$$

*ρ* is the maximum consumption rate of drug per proliferative cell voxel. The drug release rate from the ECs will be:

$$S_{c}\left(x,y,z\right)=\left\{\begin{array}{ccc} s_{c} & \text{if} & \left\{\left(x,y,z\right)\subset \text{Endothelial cell}\right\}\\ 0 & \text{if} & \left\{\left(x,y,z\right)⊄\text{Endothelial cell}\right\} \end{array}\right.$$

- **Initial and boundary conditions:**

The simulation starts with a tumor, with a diameter of approximately 65 µm, at the center of the computational domain. Initially, it is assumed that the signaling from all receptors, including the RTK receptors, is active, and the concentration of nutrients is sufficient to irrigate the cells. So, the initial and the boundary conditions are: $n{\left(x,y,z,t\right)}_{\left(x,y,z\right)\subset \text{ECs}}=s_{n}$, $n\left(x,y,z,0\right)=S_{0}=4.6\text{pg}/\text{voxel}$.

Since the secretion of VEGF is induced in the hypoxic area of the tumor, there is no VEGF concentration in the domain until a hypoxic core is formed, which means $V\left(x,y,z,0\right)=0$. In response to hypoxia, VEGF is secreted at a rate $s_{V}$ inside the tumor core.

### 2.4 Simulation algorithm

To simulate tumor growth with realistic capillary structures, the model couples multiple time and length scales. At every time step, each cell gathers information from its microenvironment to feed the simulated signaling pathways. At the intracellular level, these signals are interpreted and the model determines each cell's phenotype. The predicted cell phenotype is implemented at the cellular level, where the model calculates the Hamiltonian variation, and, based on probability values, changes are applied to the system. The model then calculates the new distribution of nutrients, VEGF and chemotherapeutic drug in the environment, which serve as inputs to the next time step. As this process is repeated, the extracellular dynamic environment controls the cells' behavior at the cellular scale.

The whole process is repeated for each pixel of the lattice at every Monte Carlo step (MCS), which can be converted into a biological scale of time. In the current model, 1 MCS represents one real‐time minute, based on the fastest cell cycle time for cell division of ∼24h.^20^, ^123^

All parameter values used in the model are listed in Table 2.

**TABLE 2:** Parameters used in the model and corresponding references

Parameter	Symbol	Value	Ref.
Nutrients diffusion equation parameters			
Nutrient diffusion constant	*D_n_*	10^3^ μm^2^/s	129
Nutrient source term	*s_n_*	8.83×10^−16^ mol/cell/s^a^	33
Nutrient consumption rate by proliferating and migrating cells	*β_P_*	5.17×10^−17^ mol/cell/s^a^	130
Nutrient consumption rate by quiescent cells	*β_Q_*	2.41×10^−17^ mol/cell/s^a^	130
Nutrient consumption rate by necrotic cells	*β_N_*	0.00 mol/cell/s	—
Drug diffusion equation parameters			
Drug diffusion constant	*D_d_*	1.5×10^3^ μm^2^/s	131
Drug source term	*s_d_*	2.55×10^−16^–5.1×10^−16^ mol/cell/s^a^	Estimated based on^131^ and ^132^ reports
Drug consumption rate by proliferating and migrating cells	*κ_P_*	9.2×10^−18^ mol/cell/s^a^	131, 132
RTK signal threshold	*T_RTK_*	4.48×10^−3^pg/pixel	133
RTK signal threshold (quiescent cells)	*T_RTK_*___ *_Q_*	8.96×10^−3^pg/pixel	Estimated
Integrin signal threshold	*T*_ITG_	0.3	Estimated
Cadherin threshold	*T*_Cadherin_	0.3	Estimated
Wnt threshold	*T*_Wnt_	0.15	Estimated
VEGF diffusion equation parameters			
VEGF diffusion constant	*D_V_*	10 μm^2^/s	134
VEGF decay	*k*	0.9375 h^−1^	134
VEGF uptake	*e*	0.001 pg/cell/s^a^	135
VEGF Source	*s_v_*	0.035 pg/pixel	6
Activation threshold	*T_v_*	0.00095 pg/pixel	20
Cellular potts model parameters			
Migrating cells elasticity	*γ*_eM_	8	Estimated
Proliferating cells elasticity	*γ*_eP_	8	136
Quiescent cells elasticity	*γ*_eQ_	2	136
EC membrane elasticity	*γ*_eEC_	0.8	20
Intracellular continuity	*α*	300	20
ECs chemotactic sensitivity	$\chi_{\sigma \_EC}$	−1.61 × 10^6^ E/conc	20
Migrating cells chemotactic sensitivity	$\chi_{\sigma \_Q}$	−1.50 × 10^6^ E/conc	Estimated
Proliferating cells chemotactic sensitivity	$\chi_{\sigma \_P}$	−1.45 × 10^6^ E/conc	Estimated
Boltzmann temperature	*T_m_*	10	Estimated
Cell–cell adhesion matrix	$J=\left[\begin{array}{cccccc} J_{\text{EC}-\text{EC}} & J_{M-\text{EC}} & J_{P-\text{EC}} & J_{Q-\text{EC}} & J_{N-\text{EC}} & J_{m-\text{EC}}\\ J_{\text{EC}-M} & J_{M-M} & J_{P-M} & J_{Q-M} & J_{N-M} & J_{m-M}\\ J_{\text{EC}-P} & J_{M-P} & J_{P-P} & J_{Q-P} & J_{N-P} & J_{m-P}\\ J_{\text{EC}-Q} & J_{M-Q} & J_{P-Q} & J_{Q-Q} & J_{N-Q} & J_{m-Q}\\ J_{\text{EC}-N} & J_{M-N} & J_{P-N} & J_{Q-N} & J_{N-N} & J_{m-N}\\ J_{\text{EC}-m} & J_{M-m} & J_{P-m} & J_{Q-m} & J_{N-m} & J_{m-m} \end{array}\right]=\left[\begin{array}{cccccc} 5 & 30 & 30 & 30 & 30 & 12\\ 30 & 8 & 8 & 8 & 10 & 12\\ 30 & 8 & 8 & 8 & 10 & 12\\ 30 & 8 & 8 & 8 & 10 & 12\\ 30 & 10 & 10 & 10 & 8 & 10\\ 12 & 12 & 12 & 12 & 10 & 66 \end{array}\right]$		

^a^ Each tumor cell has an initial volume of about 64 voxels.

### 2.5 Computational setup

Using the open‐source CompuCell3D simulation environment (http://www.compucell3d.org/), a 3D tumor vascular growth model and its response to therapy has been developed. The modeled microenvironment is a 150 × 150 × 200 lattice, equivalent to 600 × 600 × 800 μm^3^.

Initially, the simulation starts with a tumor size of ∼65μm, containing proliferating cells, surrounded by pre‐existing vessels. Nutrients are constantly diffused from the vascular network, while the diffusion of the chemotherapeutic drug is carried out according to a specific treatment protocol. Moreover, the concentration of VEGF secreted from hypoxic cells is calculated, being a driver for ECs' activation, which leads to neo‐vessel growth.

A sensitivity analysis is performed to tune the model and to identify and adjust key parameters. Hence, by varying a particular parameter at a time (and keeping fixed all the other Table 2 parameters), the main observations were listed as follow. Considering the values for the adhesion energy between cells, decreasing the *J* value leads to a higher bond that drives unrealistic cell's shape with greater tumor cell densities, while it causes an accumulation of ECs during angiogenesis, ending up in a rupture of the parent vascular structure. In contrast, increasing *J* results in a less cohesive population of tumor cells and in a separation of tip ECs from the parent vessel. In what concerns cell–matrix binding energies, the stronger bond the more elongated the cell. Values reported in Table 2 show a balance between the contact guidance and the cell–matrix adhesion energy. Assessing the compressibility properties, cell size is sensitive to *γ_e_*, which when large made the cells small, resistant to deformation and requiring more energy to grow. The results are also insensitive to the value of *T_m_* until it is increased by more than two orders of magnitude. The larger values of *T_m_*, the larger the cell membrane fluctuations.

To investigate the sensitivity of the obtained results to changes on the signaling thresholds, comparisons between numerical simulations and experimental data were performed, and the main results are the following:

- A higher activation threshold of a receptor means that the corresponding receptor is unlikely to be activated.
- Increasing the threshold for RTK receptor activation, a regulator of cell survival suppresses tumor progression.
- The E‐cadherin threshold controls contact inhibition of growth. Accordingly, for low values (<0.2) proliferation of cells is completely inhibited. For *T*_Cadherin_ ≥ 0.2, E‐cadherin regulates tumor growth, and vessels' extension velocity increases in a way insensitive to the threshold.
- The activity of Wnt signaling pathway is dependent on cadherin, and when *T*_Wnt_ ≥ 0.15, it plays a role in cell migration.
- Integrin regulates proliferating tumor cell migration toward the vessels and ECs movement toward the VEGF gradients when 0.25 ≤ *T*_ITG_ ≤ 0.3, by controlling cell–ECM connection.

To show the robustness of our 3D multiscale model, the analytical solution of the Glioblastoma (brain tumor) growth model is presented to evaluate the chemotherapy, which is accessible in the Supporting Information. Introducing the fraction of killed cells (FKCs) as a criterion for assessment of treatment efficacy, the FKCs predicted by the current model are compared with those from the analytical solution during tumor recurrence.

## 3 RESULTS

### 3.1 Cell phenotype assessment

The cells' dynamics are controlled by the signals they constantly receive from their microenvironment. Hence, a Boolean network model is employed to predict the cell phenotype from the various receptor activation states, dependent on the implemented signaling cascades (Figure 1A). The input–output map extracted from this Boolean network is presented in Figure 1B. The states' activation is represented by Boolean variables 1 and 0, corresponding to on and off switches of each component, respectively. At the top of the table are given the states of the integrin, RTK, and Wnt receptors, while the signals from E‐cadherin and tumor suppressors, APC and NF1, are indicated at the right and left of the table, respectively. The predicted cellular behavior is indicated by four Boolean variables corresponding to “cell growth,” “cell proliferation,” “apoptosis,” and “migration.” Out of the 16 theoretically possible combinations, the network only produces three biologically relevant cell phenotypes: “cell growth, proliferation, and migration” (1101), “cell apoptosis” (0010 or 0011), and “cell growth and proliferation” (1100). For instance, considering cadherin activation, case (110) represents signaling from integrin and RTK, and no signal from Wnt, in which instance the model predicts cell growth and proliferation (1100). In the table can also be observed that, in this scenario, the tumor suppressor NF1 would be able to change the cell's phenotype into the apoptotic state (0010). Here is given a summary of the major results of the signaling transduction network analysis:

- Consistent with experimental observations reported in,^137^, ^138^, ^139^, ^140^ cell apoptosis is the dominant phenotype when a disruption in the activity of either RTK or integrin receptors occurs. This is independent from E‐cadherin activity.
- In the presence of RTK and integrin, signaling from the cadherin regulates cell motility, confirming its role in cell–cell contacts.
- In the absence of Wnt signaling, cell migration can be blocked by cadherin (case 110).
- Although the reintroduction of APC into mutant cells is explored as a therapeutic strategy to drive cell apoptosis, by inhibiting pathways activated by the loss of APC, including Wnt/β‐catenin,^74^, ^75^ according to the Boolean network implemented there is no clear evidence for an APC role in apoptosis and in control of Wnt signaling.^141^, ^142^, ^143^
- In the case of main receptors' activity, the presence of NF1 impairs further progression to malignancy by inducing apoptosis. This shows a potential treatment strategy by restoring NF1, although there is no systemic therapy until now.

To incorporate the resulting mapping, it should be noted that the activation of signals from integrin, RTK, and Wnt is related to E‐cadherin activation.^144^, ^145^, ^146^, ^147^ The implementation of Wnt activity depends on the E‐cadherin loss, which stimulates canonical Wnt signaling. E‐cadherin is related to cell–cell contacts; the connection of each cell with its adjacent cells is a criterion that determines the activity of E‐cadherin receptor signaling. Integrin activity is associated with its role in cells binding to the ECM and, therefore, the connection between cells and ECM defines the activation of the integrin receptor. The activation of the RTK receptor signaling is controlled by nutrients availability, due to the role of the PI3K‐Akt pathway in the promotion of glycolysis, necessary for cell growth.^110^, ^148^ Therefore, the corresponding state is determined by the nutrient consumption rate averaged over the cell size. As a consequence, the thresholds for the turn‐on of each receptor determine the state of signal transduction pathways and allow to track cell dynamics and capture the tumor morphological changes along the process. Similarly, it has been assumed that the concentration of VEGF to activate the ECs must be above a threshold for sprouting angiogenesis initiation. Viable tumor cells can be in one of three different states: quiescent, proliferating, and migrating, with different oxygen consumption rates, according to experimental data reported by Freyer.^130^ It should be noted that the quiescent state is a distinct state from those of Figure 1A, since it is not detectable through the signaling network. Tumor cells are able to be in a quiescent slow‐growing state in regions of hypoxia and nutrient deprivation in areas far from vessels.

### 3.2 Model verification

To measure the network robustness against the fluctuations and, at the same time, investigate whether the signal transduction is independent of the initial internal states, a systematic simulation is performed for all possible 2^29^ initial combinations of states of all 29 internal components of the network described in Figure 1A. Results show that the network dynamics in all these cases converge to the final four attractors (1101, 1100, 0010, and 0011), summarized in Figure 1B, with a small difference in convergence time, confirming the high robustness of the signal transduction network. Moreover, the simulations have a strong sensitivity to transient switching of the main external signals, since any changes on inputs lead to the correspondent attractor after only a few updates.

Considering the input/output map, the final results are consistent with the experimental observations. Some important confirmations are mentioned in the last section. For instance, cell apoptosis is predicted as a cell response to the inhibition of the pathways Ras/Raf/MEK/ERK and PI3K/PTEN/Akt/mTOR. This is confirmed by various experimental results and included as a targeting pathway in developing a targeted therapy.^94^, ^149^ The model enables us to profit from this result in the treatment proposal.

**FIGURE 2:**
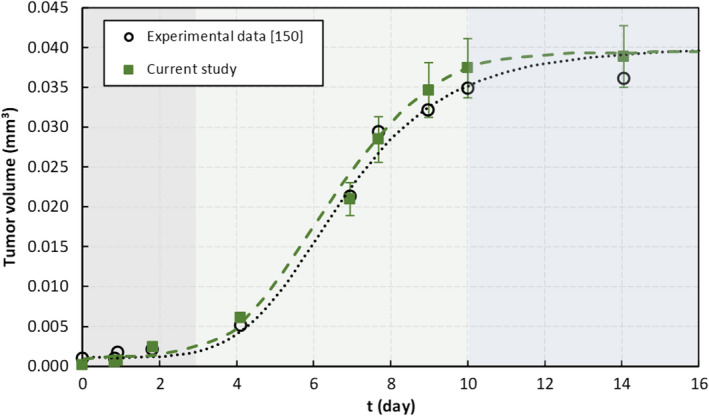
Growth curve of a tumor spheroid. Comparison between simulation results from the present work with the experimental data reported in.^150^ Error bars represent standard deviations of the mean of 5 simulations

The current multiscale model is initially compared with experimental data from a study of a long‐term 3D tumor cultivation model.^150^ The authors reported the tumor spheroid growth in a microfluidic system and measured the tumor volume evolution over time. Modeling a similar situation, the avascular growth of a spherical tumor is compared with the experimental results, in which the variation between experimental and numerical results indicates a small difference of 9% on average (Figure 2). The estimated tumor size is an average of 5 independent simulations with the same parameter set.

Accordingly, the growth pattern of the tumor from the simulation is consistent with the experimental measurements. Experiments claim that in spheroid cultures three different phases of growth can be observed. In the first phase, days 0–3, initial cell aggregation and spheroid formation at a slow rate is reported.^150^ Similarly, the numerical results imply that there is not a noticeable change in the tumor volume since the cell proliferation depends on the binding of adhesive proteins.^151^ For the small tumor size, the adhesion energy, as a regulator of contact inhibition of proliferation, is still developing and not high enough to lead to a considerable increase in cells' number. However, in the second phase, that is, days 3–10, there is an intense cell proliferation and, as a result, a fast volume increase is observed. In contrast with the first phase, the signaling from E‐cadherin controls cell–cell adhesion in the proliferation of new cells. E‐cadherin promotes tumor cell proliferation,^152^ facilitates the interaction between cells, and keeps them together, although a low expression of cell adhesion molecules leads to the loss of contact inhibition in proliferation.^62^, ^63^, ^64^ However, after day 10, proliferation slows down and the tumor volume is almost constant. This can be interpreted as a stage in which tumors are avascular and likely to still be benign. These tumors have typical spheroid shape, with a necrotic core surrounded by layers of viable proliferating and quiescent cells, as was reported in.^150^ So, there is no significant increase in tumor size in the third phase of growth. See Video S1 and Video S2 for tumor avascular growth.

### 3.3 3D tumor growth in a vascular network

**FIGURE 3:**
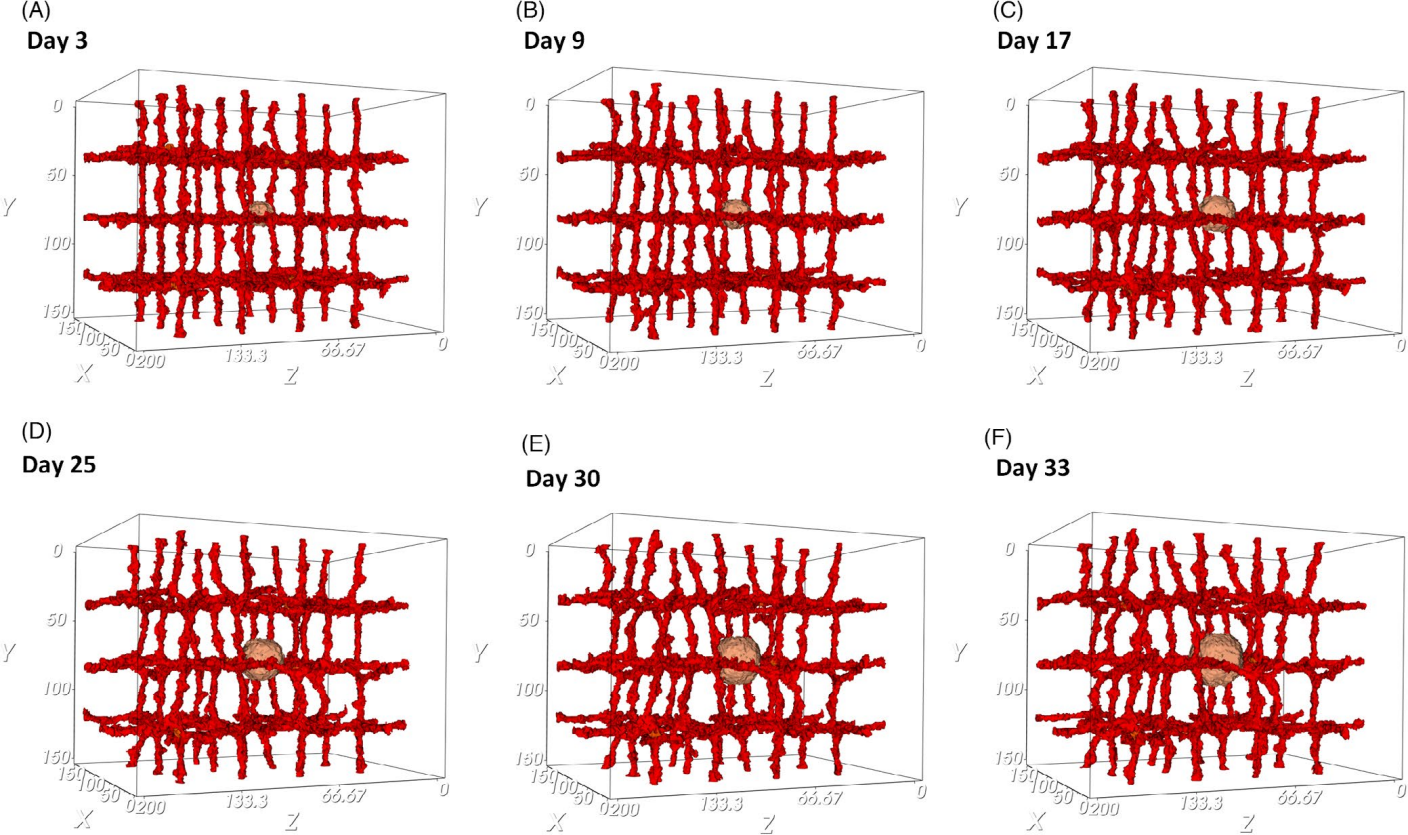
Evolution in time of an avascular tumor. (A) 3rd day of growth, the tumor starts growing, while the signaling pathways are activated and lead to growth, proliferation, and migration of cells. (B) 9th day of growth, spheroid formation is observed. (C) 17th day of growth, cell proliferation leads to a fast volume increase. (D) 25th day of growth, the tumor is constantly growing. (E) 30th day of growth, cell behavior is based on receiving signals from their environment. (F) 33rd day of growth, the tumor maintains its spherical shape

**FIGURE 4:**
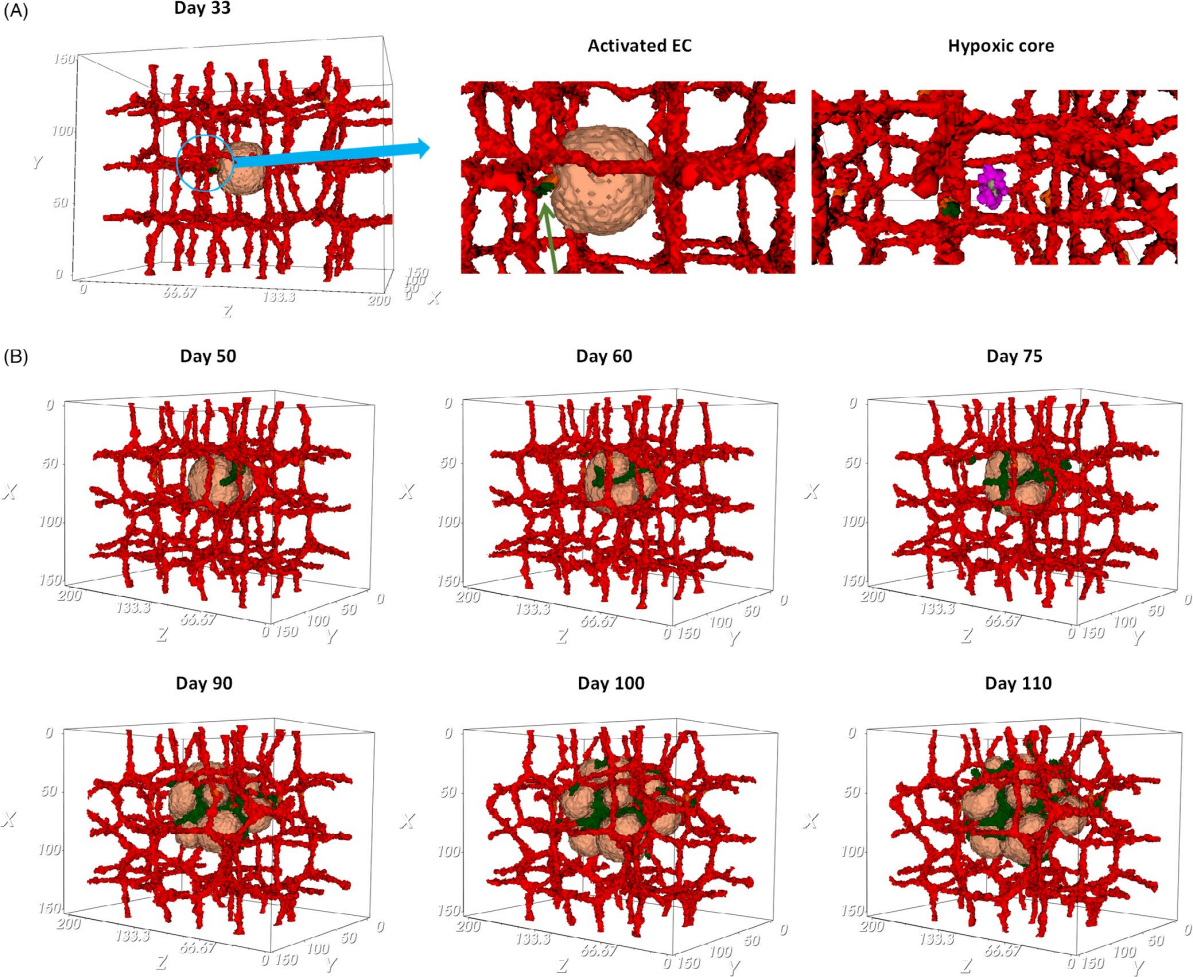
Tumor vascular growth. (A) Induction of the first activated EC (identified in green) at day 33. EC will move toward the higher VEGF concentration (zoom in) released by quiescent and necrotic cells of the tumor core (zoom in). (B) Evolution of tumor and vascular morphology during sprouting angiogenesis, days 50–110

Figure 3. shows the tumor growth in the presence of pre‐existing blood vessels at different time points. The primary capillary plexus has a regular structure, with ordered patterning that produces nutrients and releases the chemotherapeutic drug into the tissue. The exponential growth of the tumor during the avascular phase continues until day 33. In these conditions, the tumor growth cannot continue after the tumor reaches a diameter of about 150 µm (Figure 3F). Due to limited diffusion of nutrients, a hypoxic core is formed inside the tumor that induces angiogenesis by secreting VEGF. The hypoxic core consists of low oxygen concentration and cells in a quiescent state that cannot proliferate. As the tumor keeps growing, depletion of oxygen and glucose influences quiescent cells and results in the appearance of necrotic cells. Figure 4A shows the first activated EC, and quiescent and necrotic cells.

#### 3.3.1 Tumor angiogenesis

When VEGF reaches the nearest vessel, it activates ECs after a few hours. Activated ECs proliferate and move by chemotaxis along the increasing VEGF concentration. The growth rate of ECs is based on the number of VE‐cadherin junctions and new vessels form a connected network peripheral to the tumor. VE‐cadherin is a transmembrane receptor‐specific of ECs and is a main adherent junction molecule that controls the stability of EC boundaries. On the contrary, the formation of new junctions regulated by VE‐cadherin blocks ECs' response to VEGF and induces contact inhibition of cells' growth. Therefore, depending on the area of cell–cell junctions, the contact inhibited stalk cell proliferation is modeled to simulate new functional and stable capillaries. Hence, not only this guarantees vessel cohesion but also it achieves optimal vasculature growth, tuning the proportions of sprout thickness and length. As a result, activated ECs form a network of vessels around the tumor and peripheral to it (Figure 4B, in green), in response to stimulation by chemotactic factors. The induced angiogenesis reproduces tumor growth in its vascular phase, which generates new sources of nutrients for cancer cells (See Video S3). The evolution of the tumor and its morphological alterations induced by sprouting angiogenesis are shown in Figure 4B until day 110.

**FIGURE 5:**
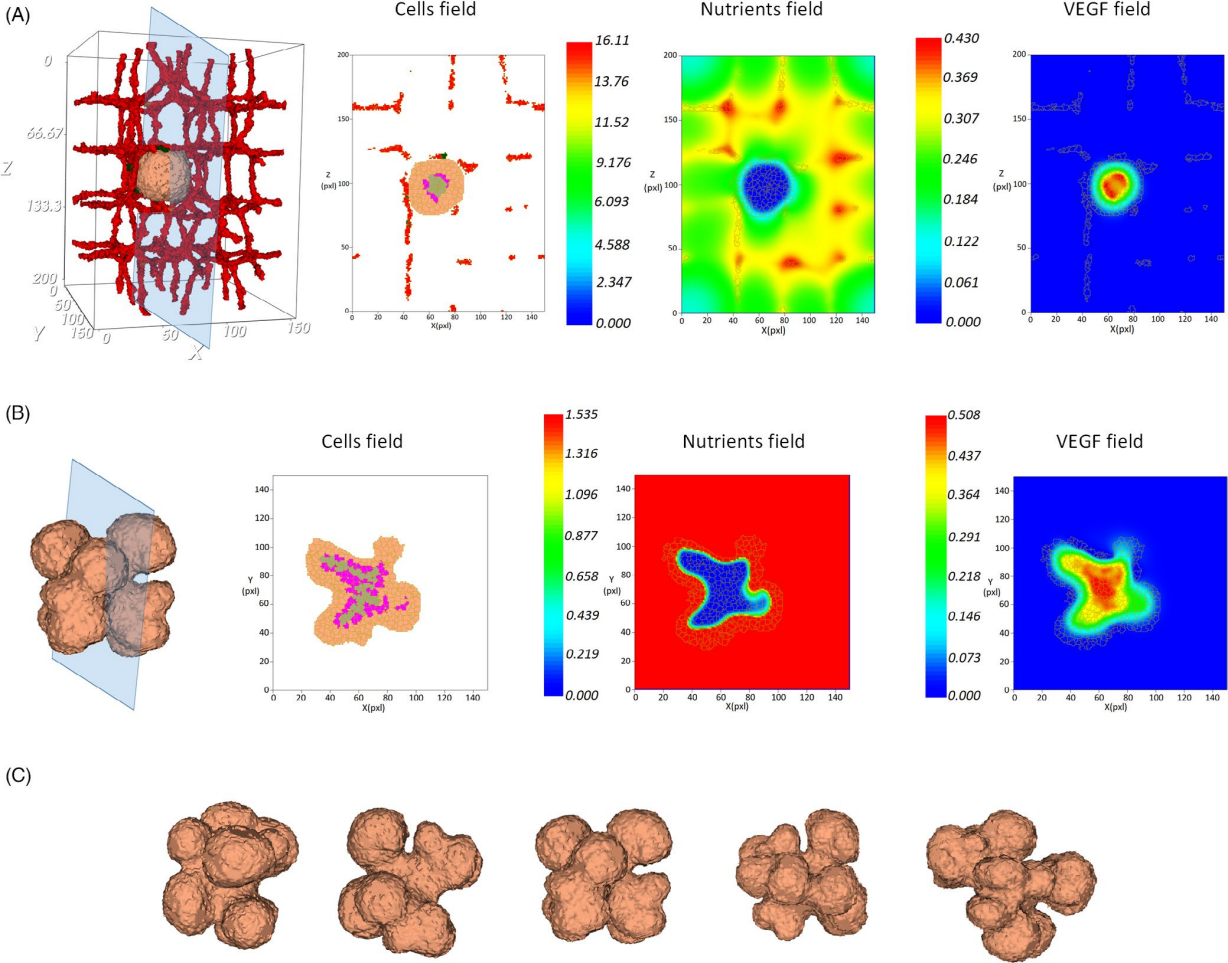
Vascularized tumor at different stages. (A) Cross section of tumor indicating cells field (proliferating and migrating cells are in orange, quiescent cells in purple, and apoptotic cells in gray), concentration of nutrients diffused from the vessels (in pg/cell/s), and distribution of VEGF produced inside the tumor tissue which decreases from the tumor inner to outer layers (in pg/cell/s). (B) Cross section of highly vascularized tumor, indicating the distribution of cells field (proliferating and migrating cells are in orange, quiescent cells in purple, and apoptotic cells in gray), nutrients field (in pg/cell/s), and VEGF field (in pg/cell/s). (C) Tumor lobulated forms on day 110 from different points of view

The model presents a clear interplay between blood vessel distribution and VEGF and nutrient concentration. New vascularization at the periphery of the tumor results in relatively higher nutrients concentration in the outer versus inner regions. Cells of the quiescent and necrotic population in the center of the tumor have a lower consumption rate. In contrast, the concentration of VEGF produced inside the tumor tissue is larger in the inner layers, which are populated by quiescent cells (Figure 5A).

As the tumor grows and the vascular network expands, the initial spherical tumor shape changes to a lobulated form. Tumor cells need to find enough room to proliferate, nevertheless, when new capillaries form and the space for cell proliferation is more confined. In fact, competition for space and resources limits tumor cell densities and influences the population distribution patterns. Cells with a high proliferation rate cause local crowding that leads to an unfavorable condition associated with limitations on resources availability and space to occupy. Hence, migration toward the vessels and to less dense locales, away from the crowding, is the most favorable situation that starts from the edge of the tumor and even can lead to movement from the interior region of the tumor. Hence, the tumor appears as a mass with a lobulated contour. The highly vascularized tumor supplies fast‐proliferating cells at the tumor periphery and lets them grow along the nearest vessels, while the poorly vascularized tumor center contains quiescent and necrotic cells that are unable to proliferate. As displayed in Figure 5B, the tumor center is a nutrient‐depleted area with a large quiescent and necrotic region. Hence, in order to access the nutrients, the tumor core releases angiogenic factors, such as VEGF at a constant rate to stimulate the growth of new capillaries. It leads to the accumulation of VEGF throughout the tumor, being the highest density inside it (Figure 5B). Figure 5C shows the highly vascularized tumor with a lobulated form on day 110 from different perspectives (See also Video S4).

### 3.4 Chemotherapy

Chemotherapy is the application of drugs that target, in general, rapidly dividing cells with the aim of killing mainly cancer cells. Despite the existence of different classes of chemotherapeutic drugs, which are based on the biochemical mechanisms of drug action, chemotherapy usually targets cell proliferation by inducing DNA damage. Hence, considering the mitotic inhibition mechanism of action in proliferating cells that leads to cell death, therapy is modeled by the distribution of drug throughout the tumor. The current model is inspired by the mechanism of cytotoxic chemotherapy drugs, such as paclitaxel, doxorubicin, and fluorouracil that affect proliferative cells, resulting in their death.

Referring to Equation 7, drug concentration in the tumor microenvironment is calculated to assess the response to chemotherapy. The response to treatment rests on tumor necrosis induced by chemotherapy, and then the level of chemotherapy‐induced necrosis is a key prognostic factor to determine a treatment plan.^153^ Histological results with high tumor necrosis following preoperative chemotherapy have a better prognosis than those with poor responses. Observations reported that in solid tumors, such as osteosarcoma and in most common childhood solid malignancies, such as Ewing's sarcoma (ES), neuroblastoma (NB), hepatoblastoma, and rhabdomyosarcoma (RMS), more than 90% of tumor chemotherapy‐induced necrosis reduces the risk of recurrence in comparison with the unsatisfactory treatment responses.

**FIGURE 6:**
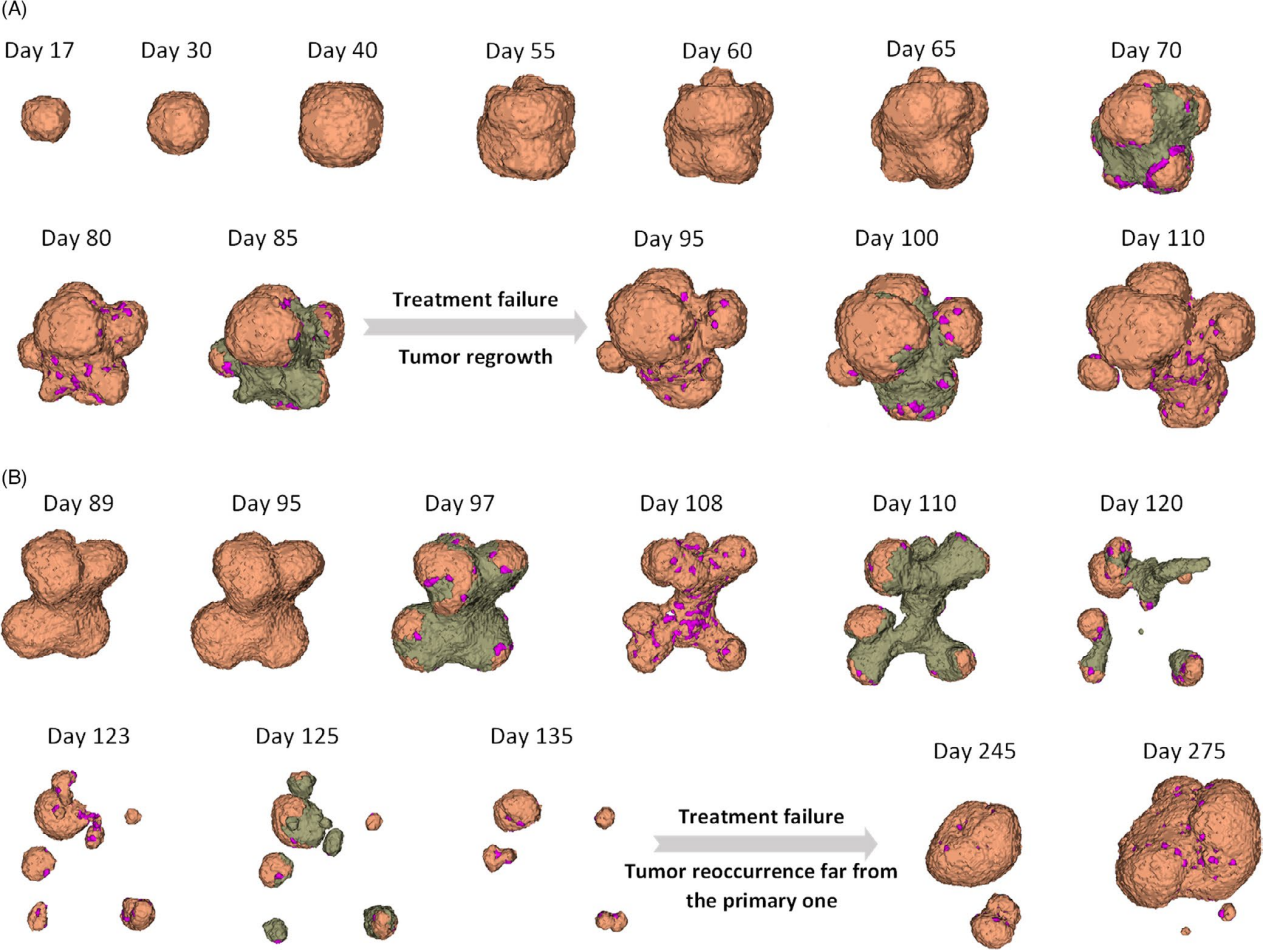
Time evolution of tumor response to chemotherapy, indicating treatment failure. (A) The tumor receives five cycles of chemotherapy at the dose of 5 µg/m^2^, from day 62, and each cycle takes about 1 week. Chemotherapy disrupts tumor development by targeting the actively dividing cells to kill and to decrease the growth rate, although the treatment ultimately fails. Proliferating and migrating cells are in orange, quiescent cells in purple, and apoptotic cells in gray. (B) Tumor response to chemotherapy, followed by growth recurrence. The tumor receives five cycles of chemotherapy at the dose of 7.5 µg/m^2^, from day 90, and each cycle takes about 1 week. Chemotherapy disrupts tumor development by targeting the actively dividing cells to kill and decrease the growth rate, although the treatment ultimately fails

Therefore, tumor responses to multiple cycles of chemotherapy are modeled, and morphological changes for two different cases are reported in Figure 6. Accordingly, in the first example, the tumor receives five cycles of chemotherapy, from day 62, and each cycle takes about 1 week (Figure 6A). Drug release induces the transition of proliferating cells into inactive quiescent cells (in purple) and finally leads to necrosis (in gray). It should be noted that necrotic cells disappear over time, as depicted in Figure 6A on days 65, 80, 95, and 110. This result is confirmed by observations in which in patients with a low percentage of necrotic cells there is an increased number of macrophages after chemotherapy, which means that the necrotic cells had already been cleared.^154^ When monitoring tumor response to therapy, there is a considerable progression of tumor from day 95 onwards, indicating treatment failure.

Once such resistance to the treatment in patients occurs, the trials are usually suspended and minor or major changes, for instance, in drug dosage and/or in the combination of drugs, route and frequency of drug administration, are introduced to the protocols. Hence, drug dosage is increased to optimize treatment efficacy (Figure 6B). In this case, treatment is implemented on day 90 while the dosage is increased by 50%. The increase in tumor necrosis leads to a favorable response to chemotherapy until day 135. However, growth rates then change unexpectedly, achieving an even faster growth rate than before the treatment. (Figure 6B, day 275).

**FIGURE 7:**
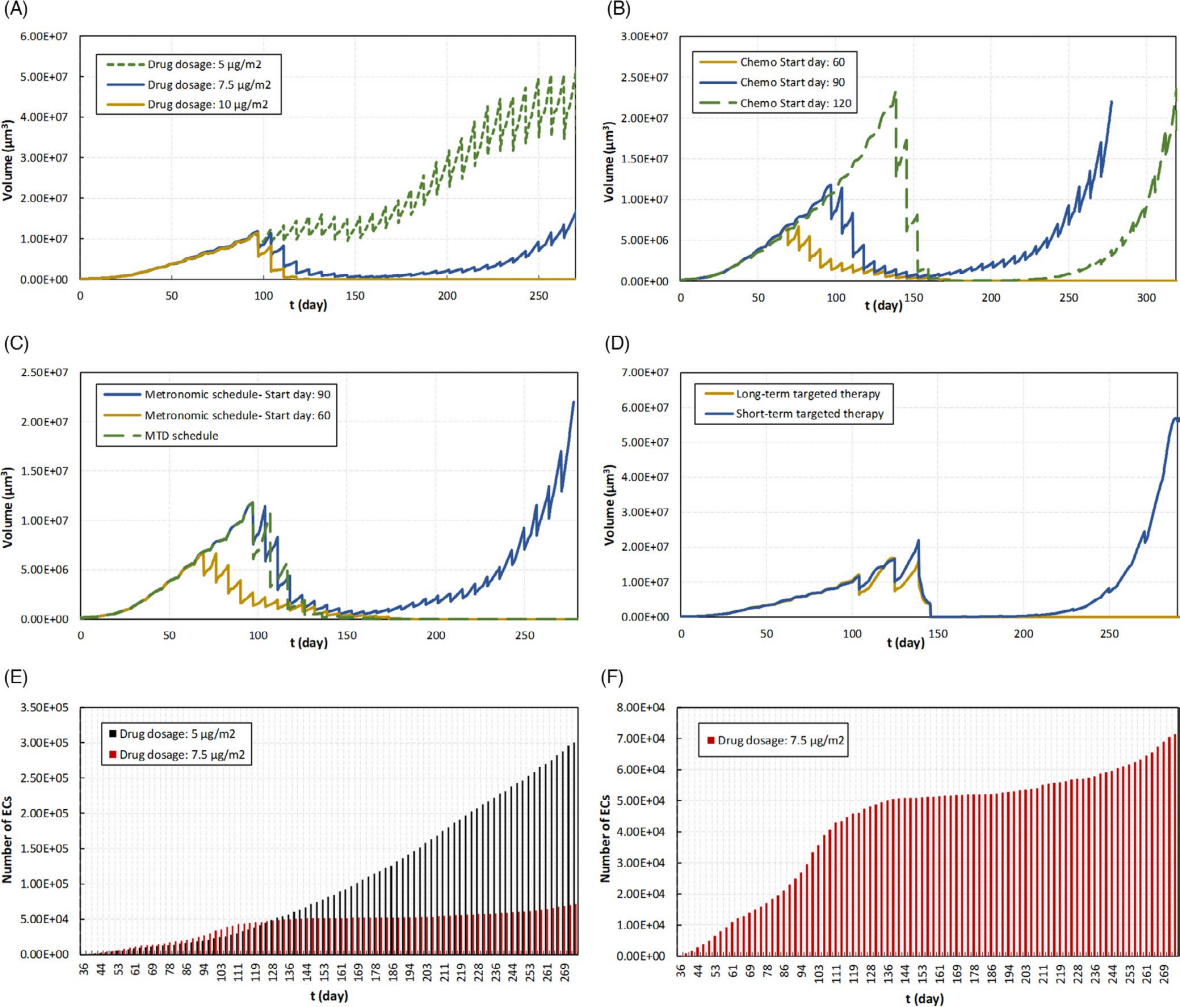
Tumor responses to various chemotherapy protocols and ECs' behavior with inadequate treatment. (A) Tumor responses to various drug dosages. The model performs a dose comparison that helps to evaluate the efficacy among different doses (i.e., 5 µg/m^2^, 7.5 µg/m^2^, and 10 µg/m^2^). Comparisons of low and high doses demonstrate that the toxicity of drug with the highest concentration (i.e., 10 µg/m^2^) eliminates the tumor after five cycles, while 7.5 µg/m^2^ at 1‐weekly intervals provides similar levels of benefit at long‐term follow‐up. However, treatment failed to stop the growth for the low dose of 5 µg/m^2^. (B) Time evolution of tumor volume for different chemotherapy initiation day (i.e., day 60, 90, or 120). Comparing a treatment initiation at day 90 with at day 120 shows that the delay in the initiation does have a significant effect on the whole process of tumor growth, although in the long term the tumor volume continues to increase throughout therapy in both cases. (C) Time evolution of tumor volume for diverse frequencies of drug administration. Drug delivery is modeled on a 10‐day periodic MTD schedule that yields a well‐responding therapy vs it is applied on a 6‐day periodic metronomic schedule. (D) Time evolution of tumor volume with a combination of chemotherapy with short‐ and long‐term targeted therapy started from day 140. Targeted drugs would enhance the efficiency of the combination treatment with chemotherapy, whether mono‐chemotherapy fails in treating the disease. (E) Change in the number of ECs in response to different chemotherapy protocols for two drug dosages (i.e., 5 µg/m^2^ and 7.5 µg/m^2^). (F) Change in the number of ECs in response to the 7.5 µg/m^2^ drug dosage, separate view

Strikingly, when the highly vascularized tumor undergoes chemotherapy‐induced necrosis, angiogenesis is the key factor that plays a pivotal role in driving the tumor aggressiveness. Tumor cells that survived the therapy start new tumor colonies far from the primary tumor and induce new vessels to nourish their resulting secondary expanding mass. Most cytotoxic chemotherapy doses are personalized according to body surface area (BSA). Hence, the values are estimated based on the transport of drug through the microvessel walls. Therefore, the model performs a dose comparison that helps to evaluate the efficacy among different doses (i.e., 5 µg/m^2^, 7.5 µg/m^2^, and 10 µg/m^2^). Comparisons of low and high doses demonstrate that the toxicity of drug with the highest concentration (i.e., 10 µg/m^2^) eliminates the tumor after five cycles, while 7.5 µg/m^2^ at 1‐weekly intervals provides similar levels of benefit at long‐term follow‐up. However, treatment failed to stop the growth for the low dose of 5 µg/m^2^ (Figure 7A).

#### 3.4.1 Chemotherapy Initiation

Several investigations reported that the delay in treatment initiation may cause recurrence of cancer and have a negative impact on overall survival.^155^, ^156^, ^157^, ^158^ By contrast, several other studies proved that there is not a clear relation between treatment initiation delays and outcomes.^159^, ^160^, ^161^, ^162^ Hence, the model analyzes the effect of delay in the initiation of treatment on outcomes. Figure 7B presents the simulation outputs for various treatment initiation dates (i.e., day 60, 90, or 120). According to these results, applying therapy at an earlier stage of tumor development leads to a remarkable reduction in its size over time and can completely eradicate the tumor. However, comparing a treatment initiation at day 90 with at day 120 shows that the delay in the initiation does have a significant effect on the whole process of tumor growth, although in the long term the tumor volume continues to increase throughout therapy in both cases. Referring to the initiation day of 120, although the therapeutic efficacy was not enough to prevent the regrowth of tumor, the tumor size is decreased significantly after the fourth cycle of chemotherapy. Since drug transport is modeled via diffusion through the endothelial cells, a well‐vascularized tumor increases drug distribution and delivery throughout the tumor. This implies tumor vasculature as a key determinant of drug transport, although the leakage rate and high permeability of new vessels enhance interstitial pressure that leads to inefficient delivery of drugs and consequent treatment failure.^39^, ^131^

#### 3.4.2 Frequency

Designing the correct drug administration frequency is important to attain the desired pharmacologic effects, reducing adverse reactions. A cytotoxic chemotherapy regimen is typically prescribed up to a maximum tolerated dose (MTD) schedule. In contrast, metronomic chemotherapy involves the frequent administration of lower doses than MTD chemotherapy to minimize the overall toxicity to the patient. Several studies investigated the effectiveness of metronomic regimens in the treatment of cancer and their impact on immune response.^163^, ^164^, ^165^, ^166^ However, more investigations are needed to optimize metronomic chemotherapy for each tumor type.^167^ Here, drug delivery is modeled on a 10‐day periodic MTD schedule that yields a well‐responding therapy and is compared to when the drug is applied on a 6‐day periodic metronomic schedule (Figure 7C).

A significant decrease in tumor size is seen as early as 4 weeks of therapy. However, results indicate that the tumor evolution changes during the metronomic schedule and tumor relapse occurs after the 10th cycle, whereas the MTD schedule seems to be more effective because of the elimination of tumor cells after the 8th cycle. Interestingly, although the total dose delivered in the 12th cycle of the 6‐day schedule (80 µg/m^2^) is equivalent to the dose delivered in the last cycle of the MTD schedule, it would not inhibit tumor growth. Simulations also show that the low‐dose metronomic chemotherapy could be effective at the early stages of cancer, eradicating all malignant tumor cells (Figure 7C). However, there is not a clear conclusion that confirms an increased effectiveness of metronomic therapy against the primary tumors at early cancer stages, although the use of combined low doses in an adjuvant therapy setting for frail elderly patients is suggested (see reviews).^167^, ^168^

### 3.5 Targeted therapies in combination with chemotherapy

Targeted therapies are based on controlling the activity of signaling pathways that regulate cell growth and survival, inhibiting proliferation and migration and even triggering apoptosis of cancer cells. To enhance the efficacy of a neoadjuvant therapy, applying targeted agents is a novel strategy that introduces targeted drugs in combination with chemotherapy.^169^, ^170^, ^171^, ^172^, ^173^ Therefore, targeting the established signaling pathways that induce cells' apoptosis and lead to tumor remission by preventing cells' proliferation and differentiation, our results on the intracellular scale are incorporated into the model. Since cell surface receptors are targets outside cells that directly regulate the downstream signals of cell cycle progression and cell death, the modulation of receptors' activity on cells' response is considered by blocking major signaling pathways. Referring to results presented in Figure 1, targeted therapy blocks some coding cases and is combined with chemotherapy in a multiscale approach. Coupling the tumor growth model with the cell‐based model of angiogenesis and the intracellular Boolean network model allows us to track the system treatment response. This is a major step toward the goal of predicting the effects of not only chemotherapy as a traditional strategy but also tumor‐targeted therapies. Experimental assays strongly suggest that the blockage of any single growth factor and inhibition of receptor tyrosine kinase or intervention on integrin‐mediated cellular adhesion limit tumor growth.^137^, ^138^, ^139^, ^140^ Tumor metastasis can be disrupted by blocking signals from E‐cadherin and related receptors to inhibit cellular migration.^144^, ^145^, ^146^, ^147^ Considering these key factors as the most promising approaches to tumor‐targeted therapies, our model focuses on intracellular signaling blockage that captures cellular apoptosis and targets the receptors that inhibit cell proliferation and migration according to the output map (Figure 1B). The applied drug dosage is controlled through the thresholds which are introduced for the activity of each receptor (Table 2). In what concerns the combination therapy, the cytotoxic chemotherapy is applied to the tumor at the same time, through Equation 9.

The results on tumor response to the different therapies are presented in Figure 7D, in which chemotherapy is applied according to the MTD strategy for a highly vascularized tumor. To better investigate the overall effect, cytotoxic mono‐chemotherapy is administered in the first two cycles and targeted therapy is applied just as the tumor is approaching its size at the beginning of the second cycle of chemotherapy. Results demonstrate a treatment success as long as targeted therapy is involved. This is consistent with the studies reporting that targeted drugs have a long‐term impact on controlling cancer and reducing the risk of disease development.^174^, ^175^ When tumor growth is stopped after 4 cycles of combination therapy, our observations indicate that as soon as signals are reactivated, tumor progression returns. This means that targeted drugs that block the major pathways of the cell progression cycle would enhance the efficiency of the combination treatment with chemotherapy, whether chemotherapy alone may fail in treating the disease.

In order to investigate the new vasculature behavior, a comparison of tumor response to two different chemotherapy protocols is presented (Figure 7E‐F). The first case considers applying the low dose of 5 µg/m^2^ at an early stage of tumor growth but it leads to treatment failure. Under these conditions, endothelial cells are rapidly expanding. In the second case, a dose of 7.5 µg/m^2^ is applied on the 90th day of tumor growth, yet it was unable to avoid tumor reoccurrence after 8 cycles. In a similar way to a previous case, the successful treatment, the growth of new arteries also stopped, to some extent, and no significant change in the number of endothelial cells was reported; however, with tumor recurrence, the angiogenic rate increased (Figure 7F).

## 4 DISCUSSION

In this study, mathematical and computational modeling methods for simulation of tumor growth and angiogenesis are used to explore opportunities in the development and testing of novel treatment strategies, including targeted therapies. The control of the signals involved in cell proliferation and, even more importantly, in cell apoptosis, is still a challenge in tumor reduction and/or elimination. Here, a multiscale model is presented to test this explicit aim in order to clarify how the signaling transduction operates and affects important tumor development processes. In addition, considering the mechanisms of cytotoxic drug usage, chemotherapy is modeled by solving the distribution of a drug throughout the tumor.

A three‐dimensional cell‐based model is developed, in which cell dynamics is estimated from a cellular model describing the interactions between cells and the ECM; at the intracellular scale, the model surveys the signal transduction network, determining the cell state evolution effected by the extracellular dynamics. Therefore, the avascular and vascular growth of a tumor is simulated in the presence of pre‐existing blood vessels. The comparison of numerical results with experimental observations shows a good agreement in the growth stages of the avascular tumor, with a small 9%difference in tumor size. The growth curve is divided into three phases, and it depends on the adhesion energy, including the E‐cadherin and integrin‐mediated cellular adhesion and signaling from growth factors that control the growth rate. As the tumor grows and the vascular network expands, the initial spherical tumor shape changes into a lobulated form. Limitations on resources and space cause cell migration toward less dense locales, avoiding over‐crowd regions. As a result, the geometry of the tumor is more irregular and disordered than the avascular tumor, and it reflects a more complex phenomenon that cannot be reproduced in 2D modeling.

Our research reveals the capability of a multiscale and three‐dimensional numerical simulation of tumor progression to explore the outcomes of drug treatment. 2D models yield valuable insights into the growth and dissemination of tumors, tumor‐induced angiogenesis, and vascular remodeling. As a matter of fact, the 2D assumptions are indeed acceptable when tumors are either approximately flat or when they have important symmetries (e.g., when they are spherically symmetric). Although modeling in 2D is an attractive alternative to 3D calculations, as it requires notably less computational resources, in the context of solving mechanical forces that mediate cell shape and orientation, the 2D hypothesis might not be sufficiently accurate in predicting tumor behavior as a growing mass. Effective mechanical forces induced by tumor cells during their movement and migration are exerted on the surrounding endothelial cells and the ECM. Intercellular adhesion forces, associated with the chemotactic and tractional forces, simultaneously regulate cell shape. Consequently, cells deform dynamically, inducing a variety of cellular processes. The presented 3D multiscale modeling improves the results of the simulation of tumor growth and related events by describing the relationship between cell function and shape based on the forces that are applied in three‐dimensional space.

The prediction of the 3D tumor geometry is fairly consistent with the real tumors, which are characterized by an irregular and disordered shape. While the 2D simulations are not able to capture these irregular deformations, they are extensively employed to simulate the process in a simplified way. The 3D version captures a tumor surrounded by a dense 3D vascular network that not only transports nutrients to the tumor tissue, but also drugs. In addition, cells have more space to move and, hence, more ease in proliferating and migrating when the local drug concentration increases. Three‐dimensional simulations can capture this effect by predicting irregular tumor shapes. The highly vascularized tumor supplies oxygen and nutrients to fast‐proliferating cells at the tumor periphery, and highly motile cells tend to move toward the nearest vessels and grow along them.

In what concerns the modeling of cytotoxic chemotherapy, tumor responses to multiple cycles of chemotherapy are simulated, including treatment failure, relapse at a distance from the primary tumor, and effective therapy. Different protocols aiming at treatment efficacy optimization have been investigated. The model performs a dose comparison that helps to evaluate the dosing efficacy. Moreover, analyzing the effect on outcome of a delay in chemotherapy initiation indicates that applying therapy at the earliest stage of tumor development leads to a remarkable reduction in its size over time. In highly vascularized tumors, representing high‐grade cancers, the initiation delay does not guarantee the treatment success since tumor eradication has not been observed. However, a significant effect on the long‐term prospects is obtained, which reflects the longer period of tumor dormancy (76 days), and postpones recurrence of tumor for more than a month (40 days).

Since drug administration frequency is important to attain the desired pharmacologic effects, and to reduce adverse reactions, the model compares the MTD schedule of chemotherapy with the metronomic regimen. Results show that low‐dose metronomic chemotherapy can be effective at the earlier stages of cancer, eradicating all malignant tumor cells.

Anti‐tumor and ECM‐targeted strategies in novel cancer treatments are based on controlling the activity of signaling pathways that regulate cell growth and survival. The current study introduces a novel technique, in three‐dimensional mathematical modeling of targeted therapies, by blocking major signaling pathways. Modeling targeted therapy in combination with chemotherapy, results show treatment success with a long‐term inclusion of a targeted drug, while mono‐chemotherapy may fail.

## CONFLICT OF INTEREST

The authors have declared that no competing interests exist.

## AUTHOR CONTRIBUTIONS

Sahar Jafari Nivlouei, M. Soltani, and João Carvalho conceived of the presented idea. Sahar Jafari Nivlouei developed the theory and performed the computations. João Carvalho, M. Soltani, Mohammad Reza Salimpour, and Ebrahim Shirani supervised the findings of this work, and Rui Travasso aided in interpreting the results. All authors discussed the results and contributed to the final manuscript. The original draft was written by Sahar Jafari Nivlouei, and its review and editing was done by João Carvalho, M. Soltani, Rui Travasso, and Sahar Jafari Nivlouei.

## Supporting information

Supplementary Material

Video S1

Video S2

Video S3

Video S4

Supplementary Material

## Data Availability

Any data generated in this study are available from the corresponding author upon reasonable request.
