# Supplementary material for: A multiscale cell‐based model of tumor growth for chemotherapy assessment and tumor‐targeted therapy through a 3D computational approach
Source: Cell Prolif. 2022 Feb 7;55(3):e13187. doi: 10.1111/cpr.13187 (PMC8891571; doi:10.1111/cpr.13187)
Supplement: Supplementary file 1 — Supplementary Material [file CPR-55-e13187-s003.docx]

**Analytical solution**

**Glioblastoma (brain tumor) growth model**

Glioblastomas (gliomas) are known as the most aggressive brain tumors with a median life of 9–12 months from detection [1]. Here we consider recurrence phase of growth during the chemotherapy at the removal stage. Considering the brain to be homogeneous, the mathematical model encompasses the two key elements of net proliferation rate of cancer cells and their diffusion through the field which means:

| (1) | $\frac{\partial c}{\partial t}=\nabla\cdot\left(D(\bar{x})\nabla c\right)+\rho c$ |
| --- | --- |

Equation (1) quantifies the rate of change of tumor cell density $c(\bar{x},t)$ in cells/mm^3^, at time *t,* as a function of the position in the brain $(\bar{x})$. $D(\bar{x})$ denotes diffusion coefficient of motile cancer cells measured in mm^2^/day and $\rho$ represents the net proliferation rate of cells.

According to the experimental observations, the migration of glioma cells is quicker in the white matter of human brain than the grey matter, which makes *D* a space dependence measure of the motility of glioma cells. So, inhomogeneous $D(\bar{x})$ for white matter, grey matter and CSF, is $D_{w}$ and $D_{g}$ and $D_{CSF},$ respectively. Studies on homogeneous and inhomogeneous brain-based models revealed that there is not a considerable difference for average value of its diffusion coefficient [2]. Hence, homogeneous model is used to simplify the procedure. It is assumed that there is a zero flux of cells at the boundaries of brain $\partialΩ$:

| (2) | $n\cdot\nabla c=0\text{ on }\partial\Omega$ |
| --- | --- |

Before diffusion starts, initial distribution of tumor cells at $t=0$, is considered the ratio of 4000 cells/mm^3^, which is defined as $c=f(\bar{x},0)$ at time zero.

**Homogeneous Drug Delivery**

To model drug delivery process, chemotherapy is introduced as a loss term to equation 1. Therefore, $G(t)$ is temporal term representing the amount of therapy at time, $t$ , as:

| (3) | $\frac{\partial c}{\partial t}=\nabla\cdot\left(D(\bar{x})\nabla c\right)+\rho c-G(t)c\text{ for }t>t_{c}\text{ where }G(t)=\left\{\begin{matrix} J,\text{ }t\in T_{on}-\text{Chemotherapy on} \\ 0,\text{ }t\in T_{off}-\text{Chemotherapy off} \end{matrix}\right.$ |
| --- | --- |

Where chemotherapy starts at $t_{c}$ with the value of *J*. The cycle of chemotherapy,$t_{\mathrm{cycle}}$, consists of the time of the drug administration,$t_{\mathrm{administration}}$ and a waiting period, $t_{\mathrm{waiting}}$. As a consequence, the periodic time at which the treatment is applied to the model will be:

$$\begin{matrix} T_{on}=\left\{\left[nt_{\text{cycle}}+t_{\text{c}},nt_{\text{cycle}}+t_{\text{adminestration}}+t_{\text{c}}\right],\text{ }n=0,1,...,N_{\text{cycles}}\right\}, \\ T_{off}=\left\{\left[nt_{\text{cycle}}+t_{\text{adminestration}}+t_{c},(n+1)t_{\text{cycle}}+t_{\text{c}}\right],\text{ }n=0,1,...,N_{\text{cycles}}\right\}. \end{matrix}$$

To solve the equation 3, a symmetric spherical tumor is considered with a constant diffusion coefficient reported in which the equation (3) becomes:

| (4) | $\frac{\partial c}{\partial t}=D\left[\frac{\partial^{2}c}{\partial r^{2}}+\frac{2}{r}\frac{\partial c}{\partial r}\right]+\left[\rho-G(t)\right]c\text{ for }t>t_{c}\text{ }$ |
| --- | --- |

**Analytical Solution**

Non-dimensional variables are introduced as following:

$$\begin{matrix} r^{*}=\sqrt{\frac{\rho}{D}}r\text{ },\text{ }t^{*}=\rho(t-t_{c})\text{ } \\ c^{*}(r,t)=\frac{1}{N_{0}}\frac{D}{\rho}c\left(\sqrt{\frac{\rho}{D}r},\rho(t-t_{c})\text{ }\right) \end{matrix}$$

It should be noted that represents initial distribution of tumor cells when the chemotherapy starts, at $t=t_{c}\text{ }$. Hence, which implies:

Defining $c^{*}(r^{*},t^{*})=v(t^{*})w(r^{*},t^{*})$, the following equations are satisfied:

Solving $v(t^{*})$and $w(r^{*},t^{*})$:

Finally, the dimensional form would be:

$$c=\frac{N_{0}}{8\left(\pi Dt\right)^{3/2}}\exp\left[-\frac{r^{2}}{4Dt}+\left[t\left(\rho-\left\langle G(t)\right\rangle\right)\right]\right]$$

**Tumor evolution**

Fig S1 shows the local distribution of cancer cells on days 100, 200 and 250 of glioma growth. Accordingly, cell density rate increases over the time, whereby the maximum concentration of cells is increases by 50% at the 250^th^ day. Parameter values are given in table 1.

| **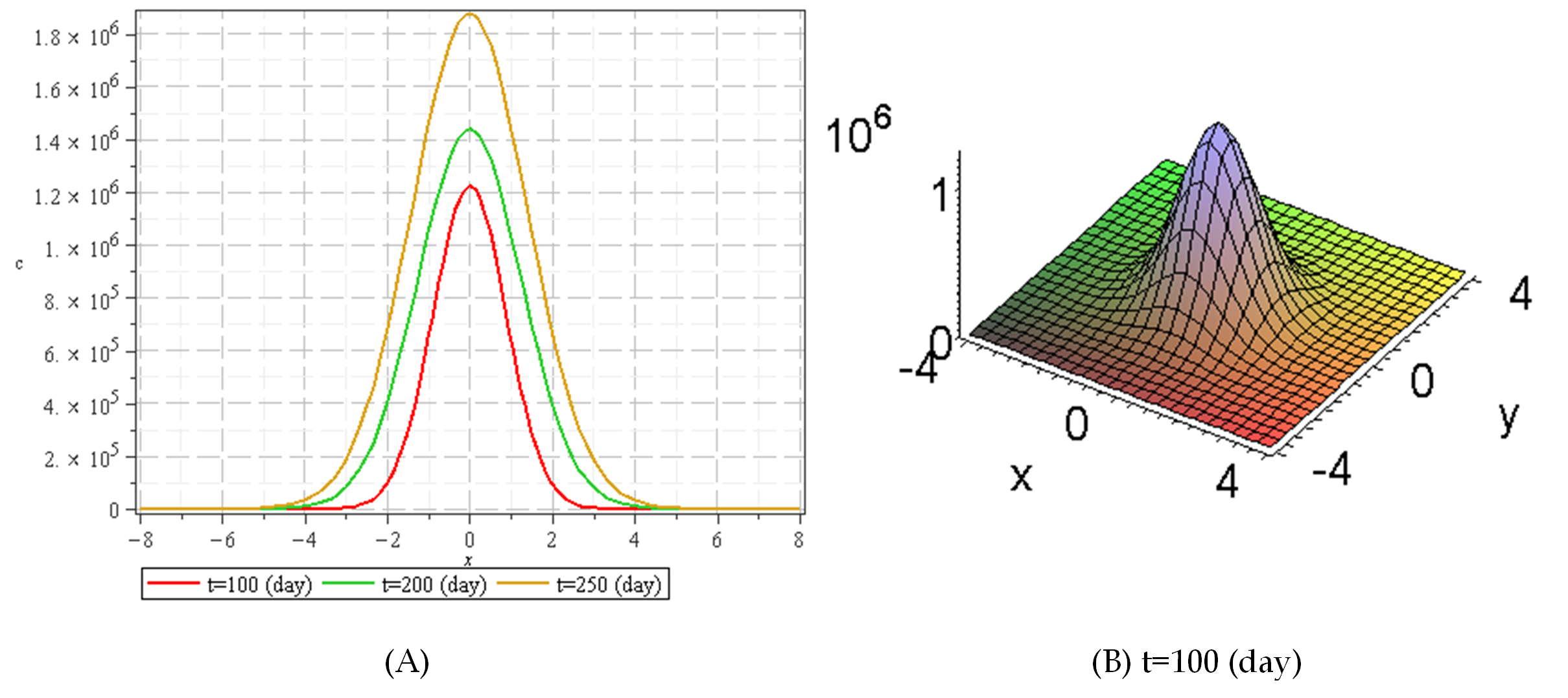** |
| --- |
| **Fig S1.** (A) Concentration of tumor cell on different days. (B) 3D plot of cancer cell distribution on day 100. |

Decreasing of tumor detectable size determines the effectiveness of chemotherapy. The detectable radius of tumor at the smallest level of cancer is a function of time that is defined by the tumor cell density. Therefore, assuming the smallest cell density as a threshold of cancer detection which is denoted by $c^{*}$, the detectable volume of tumor is:

| (5) | $r^{*}=2t\sqrt{D}\sqrt{\left(\rho-\left\langle G(t)\right\rangle\right)-\frac{1}{t}\ln\left[\frac{c^{*}}{N_{0}}\left(4\pi Dt\right)^{3/2}\right]}$ |
| --- | --- |

To investigate the effectiveness of treatment, the evolution of detectable tumor volume for various chemotherapy strengths is presented in Fig S2. The growth of tumor without applying the drug is described by the curve J=0, meanwhile, curves with different J values show drug administration in tumor. Accordingly, tumor keeps growing even in the relatively effective chemotherapy (J=6) for larger time expressing recurrence of growth, while the detectable volume approaches to zero at the primary stages of treatment for short time.

| 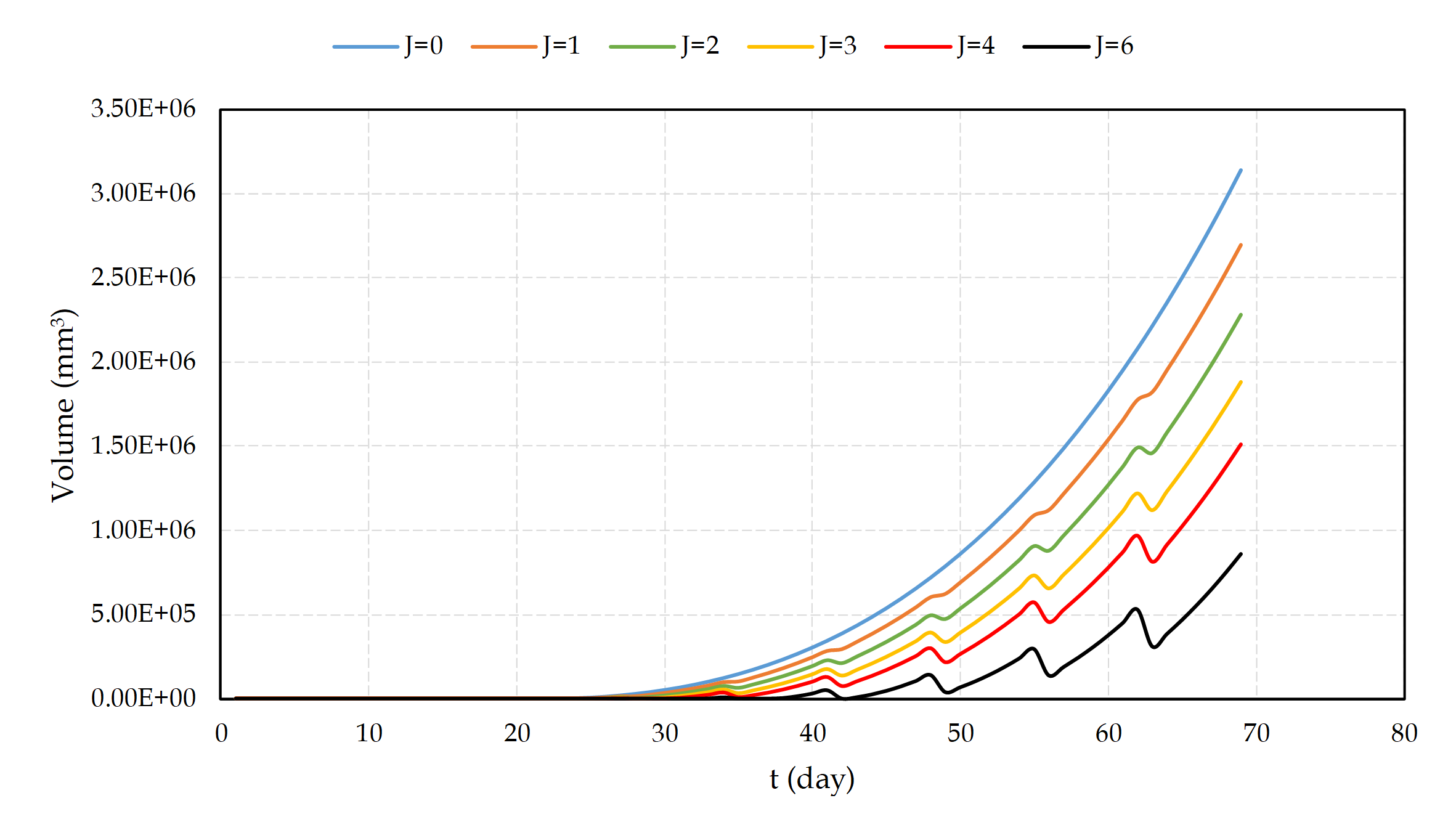 |
| --- |
| **Fig S2.** Tumor detectable volume changes during various strength of chemotherapy |

**Table. 1.** List of parameter values

| Parameter | Symbol | Value | Ref. |
| --- | --- | --- | --- |
| Growth rate | $\rho$ | $0.012\text{ 1/day}$ | [3] |
| Diffusion coefficient (average value) | $D$ | $0.0013\text{ cm}^{2}\text{/day}$ | [3] |
| The detection level | $c^{*}$ | $4000\text{ cell/mm}^{3}$ | [3] |
| Length of chemotherapy per cycle | $t_{\text{administration}}$ | $1\text{ day}$ | - |
| Length of chemotherapy cycle | $t_{\text{cycle}}$ | $1\text{ week}$ | - |

**Comparison with numerical results**

The efficacy of anticancer drugs, can be described by the fraction of killed cells (FKCs) which is calculated as follows[4]:

| (6) | $FKCs=1-\frac{N}{N_{0}}$ |
| --- | --- |

where $\frac{N}{N_{0}}$ is the fraction of survived cells after the chemotherapy. The tumor cells’ regrowth after each cycle of treatment is important to predict the cancer response. Using FKCs as a criterion for assessment of treatment efficacy, we compare the numerical results (from the multiscale model) of tumor recurrence for the J=6, in Fig S3.

Results show more than 80% and 90% of chemotherapy efficiency in the first cycle of treatment in numerical simulations and analytical solution, respectively. Since the tumor continues to grow, the FKCs decreases over time and its slope changes smoothly in the most effective condition for large time. As is clear, the results of the numerical study and analytical solution predict the FKCs in a similar trend, indicating high robustness of our multiscale model.

| 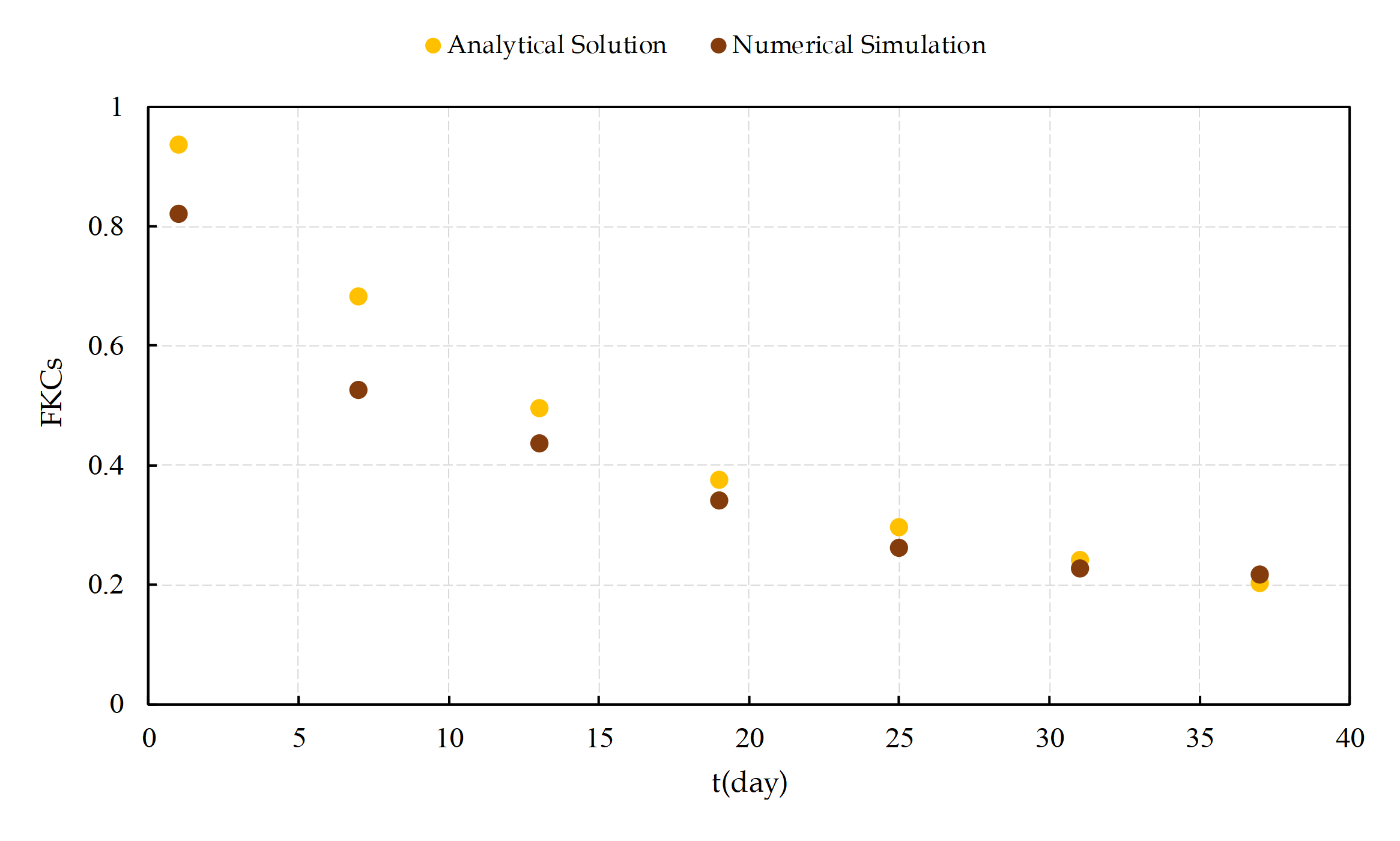 |
| --- |
| **Fig S3.** Assessment of chemotherapy efficacy using FKCs. Comparison between simulation results and analytical solution. |

**References**

[1] P. Macklin, and J. S. Lowengrub, “A new ghost cell/level set method for moving boundary problems: application to tumor growth,” *Journal of scientific computing,* vol. 35, no. 2-3, pp. 266-299, 2008.

[2] G. S. Stamatakos, and S. G. Giatili, “A Numerical Handling of the Boundary Conditions Imposed by the Skull on an Inhomogeneous Diffusion-Reaction Model of Glioblastoma Invasion Into the Brain: Clinical Validation Aspects,” *Cancer Informatics,* vol. 16, pp. 1176935116684824, 2017/01/01, 2017.

[3] K. R. Swanson, C. Bridge, J. Murray, and E. C. Alvord Jr, “Virtual and real brain tumors: using mathematical modeling to quantify glioma growth and invasion,” *Journal of the neurological sciences,* vol. 216, no. 1, pp. 1-10, 2003.

[4] F. Mpekris, S. Angeli, A. P. Pirentis, and T. Stylianopoulos, “Stress-mediated progression of solid tumors: effect of mechanical stress on tissue oxygenation, cancer cell proliferation, and drug delivery,” *Biomechanics and modeling in mechanobiology,* vol. 14, no. 6, pp. 1391-1402, 2015.
