# Supplementary material for: A multiscale cell‐based model of tumor growth for chemotherapy assessment and tumor‐targeted therapy through a 3D computational approach
Source: Cell Prolif. 2022 Feb 7;55(3):e13187. doi: 10.1111/cpr.13187 (PMC8891571; doi:10.1111/cpr.13187)
Supplement: Supplementary file 6 — Supplementary Material [file CPR-55-e13187-s001.docx]

**Supporting Information**

An analytical solution is presented, describing a glioblastoma growth model and homogeneous drug delivery. A comparison between numerical results and an exact solution of chemotherapy efficiency in tumor relapse is provided. The treatment efficacy is described by the fraction of killed cells (FKCs).

Movie S1: tumor avascular growth

Movie S2: tumor avascular growth_ Final perspective

Movie S3: tumor vascular growth

Movie S4: tumor vascular growth_ Final perspective
